# Hydrolyzable Bio‐Based Bisphenols Enabled by the Tishchenko Reaction for Polyurethane Vitrimers with Closed‐Loop Recyclability

**DOI:** 10.1002/advs.202503152

**Published:** 2025-04-11

**Authors:** Jiewen Wang, Hongru Qiang, Rong Huang, Dan Zhao, Zihan Tong, Zhen Fan, Jianzhong Du, Yunqing Zhu

**Affiliations:** ^1^ Department of Polymeric Materials School of Materials Science and Engineering Tongji University Shanghai 201804 P. R. China; ^2^ Department of Gynaecology and Obstetrics Shanghai Key Laboratory of Anesthesiology and Brain Functional Modulation Clinical Research Center for Anesthesiology and Perioperative Medicine Translational Research Institute of Brain and Brain‐Like Intelligence Shanghai Fourth People's Hospital School of Medicine Tongji University Shanghai 200434 P. R. China; ^3^ Key Laboratory of Advanced Civil Engineering Materials of Ministry of Education School of Materials Science and Engineering Tongji University 4800 Caoan Road Shanghai 201804 P. R. China; ^4^ School of Materials Science and Engineering East China University of Science and Technology Shanghai 200237 P. R. China

**Keywords:** biobased bisphenols, closed‐loop recyclability, green chemistry, renewable resources, vitrimer

## Abstract

Polyurethane (PU) is a cornerstone of modern materials science, yet its reliance on petroleum‐based precursors and the limited recyclability of conventional formulations pose significant environmental challenges. In this study, a fully bio‐based polyurethane vitrimer system is developed enabled by a dual‐function SmI_2_‐mediated strategy that integrates Tishchenko coupling and phenol deprotection in a single step, simplifying the synthesis of bio‐based bisphenols with 100% atom utilization. These bisphenols introduce hydrolyzable ester bonds, allowing for complete degradation within ≈3 d (representative model), providing an efficient and eco‐friendly end‐of‐life solution. This approach offers a sustainable alternative to conventional bisphenol A (BPA). Moreover, by leveraging the electronic effects of bio‐based bisphenols, the dissociation temperature of phenol‐carbamate bonds can be widely tuned (≈70–120 °C), endowing the resulting Covalent Adaptable Network (CAN) PUs with excellent reprocessability, closed‐loop recyclability, and reconfigurable shape memory capability. Furthermore, the aromatic and ester‐rich structure enhances thermomechanical performance, yielding tensile strengths up to 33 MPa, elongations at break exceeding 400%, and toughness reaching 30 MJ m^−^^3^, surpassing most sustainable PUs. This work pioneers a scalable and fully bio‐based PU vitrimer platform with tunable performance, recyclability, and sustainable degradability, offering a compelling alternative to traditional thermosets and thermoplastics for next‐generation green materials.

## Graphical abstract

This study employs the Tishchenko coupling reaction to obtain bio‐based bisphenols containing degradable ester bonds with 100% atom utilization. Based on these bio‐based bisphenols, a fully bio‐based polyurethane Covalent Adaptable Networks are constructed, while simultaneously endowing the material with closed‐loop recyclability, degradability, and reconfigurable topology properties.

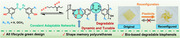

## 1 Introduction

Polyurethane (PU) materials are widely used in coatings, adhesives, elastomers, and foams, owing to their excellent thermal and mechanical properties.^[^ ^1^, ^2^ ^]^ However, the reliance on petroleum‐based raw materials and the lack of effective end‐of‐life disposal methods have presented significant environmental challenges. Most conventional PUs are either incinerated, releasing greenhouse gases, or disposed of in landfills, leading to soil pollution.^[^ ^3^, ^4^ ^]^ Additionally, traditional thermosetting PUs, due to their permanent crosslinking networks, are inherently difficult to degrade, further exacerbating environmental concerns.^[^ ^5^ ^]^ To address these issues, it is essential to develop sustainable alternatives that not only utilize renewable resources but also exhibit degradable and environmentally friendly lifecycle characteristics.

One promising approach to achieving this goal involves the incorporation of Covalent Adaptable Networks (CANs) into polyurethane systems.^[^ ^6^, ^7^ ^]^ CANs rely on dynamic covalent bonds, which can reversibly dissociate and reform under external stimuli (such as heat or light).^[^ ^8^, ^9^ ^]^ This unique property bridges the gap between thermosetting and thermoplastic materials, offering reprocessability and recyclability while maintaining the performance advantages of thermosets. Many dynamic covalent bonds have been developed, such as transesterifications,^[^ ^10^, ^11^ ^]^ disulfide bonds,^[^ ^12^, ^13^ ^]^ imine bonds,^[^ ^14^, ^15^ ^]^ acetal bonds,^[^ ^16^, ^17^ ^]^ dithioacetal bonds,^[^ ^18^, ^19^ ^]^ Diels‐Alder adduct,^[^ ^20^, ^21^ ^]^ phenol–carbamates.^[^ ^22^ ^]^ Among these, phenol‐carbamate bonds, formed between isocyanates and phenols, exhibit dynamic properties at moderate temperatures (below 120 °C),^[^ ^22^ ^]^ making them particularly suitable for the preparation of crosslinked polyurethane networks with enhanced recyclability.

At the same time, the widespread use of petroleum‐derived bisphenol A (BPA) in polymer synthesis has raised increasing concerns due to its environmental persistence and potential health risks.^[^ ^23^, ^24^ ^]^ BPA is non‐degradable in natural environments, contributing to long‐term pollution, and has been linked to endocrine disruption, leading to stricter regulations and a pressing demand for safer alternatives.^[^ ^24^, ^25^, ^26^, ^27^, ^28^ ^]^ Furthermore, the depletion of fossil resources and the need to reduce carbon emissions have made replacing BPA with sustainable, bio‐based substitutes an urgent priority.^[^ ^29^, ^30^ ^]^ Developing renewable and degradable bisphenols with comparable or superior performance to BPA is crucial for advancing green and sustainable materials.

Bio‐based aromatic aldehydes represent a promising class of sustainable building blocks for high‐performance polymers, as they retain the structural advantages of petroleum‐derived bisphenols while enabling degradability through chemical modifications.^[^ ^31^, ^32^ ^]^ These aldehydes can be sourced from renewable feedstocks, including lignin depolymerization, which produces benzaldehyde derivatives such as p‐hydroxybenzaldehyde, vanillin, and syringaldehyde.^[^ ^33^, ^34^, ^35^, ^36^ ^]^ Their intrinsic methoxylation patterns (─OCH₃ at C3/C5 positions) and phenolic −OH groups provide structural versatility, allowing for tailored electronic properties and chemical reactivity in polymer applications.^[^ ^37^ ^]^ Despite their potential, the incorporation of degradable functionalities (e.g., ester bonds) into bio‐based bisphenols remains underexplored. Expanding this approach is essential for developing fully bio‐based, degradable crosslinked PU networks that align with the principles of sustainable materials innovation.^[^ ^35^, ^38^, ^39^ ^]^

**Scheme 1:**
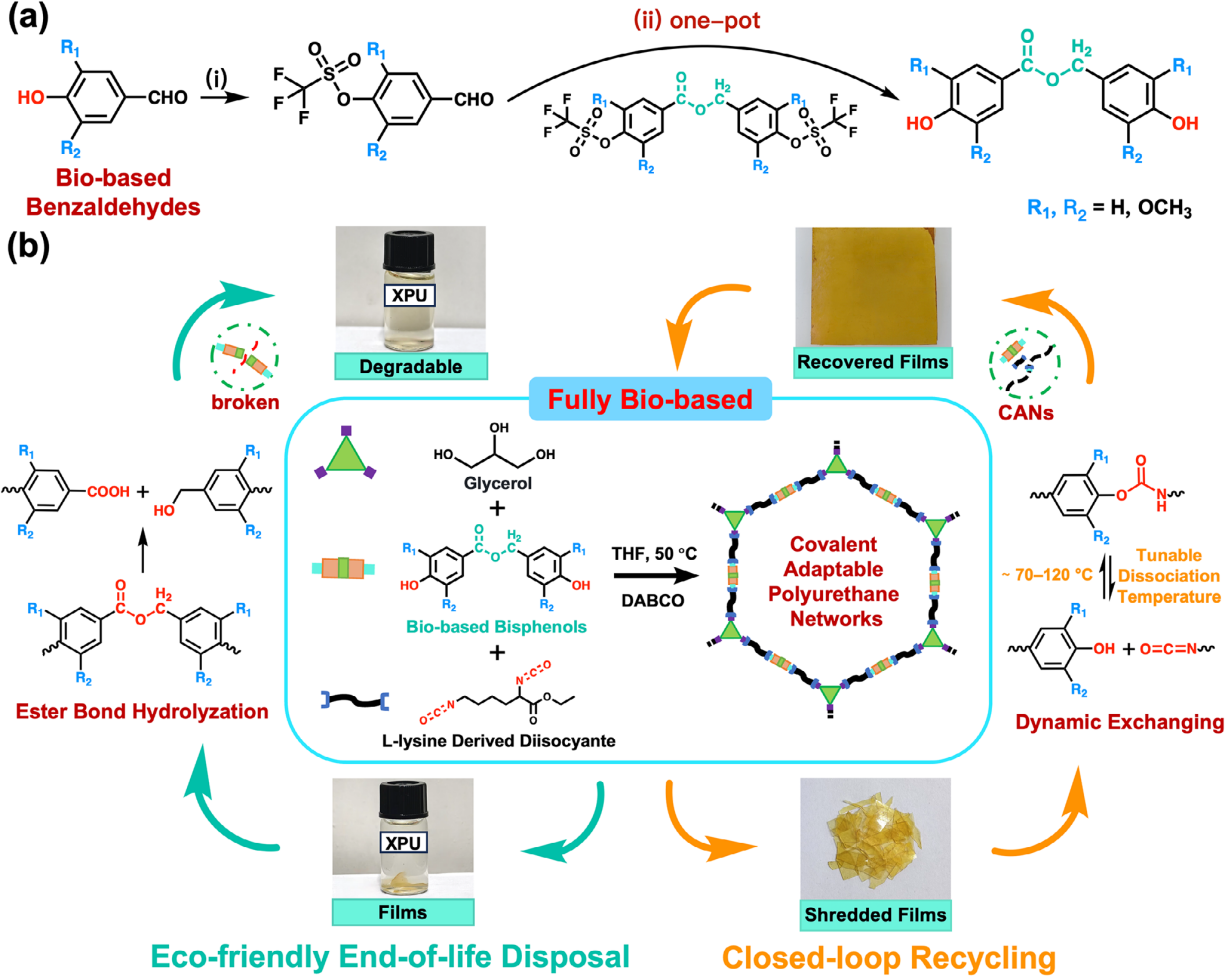
a) Synthetic route of bio‐based bisphenols. Reagents and conditions: (i) 1.0 equiv. of vanillin, 1.2 equiv. of trifluoromethanesulfonic anhydride, 3.4 equiv. of pyridine, DCM/DMF, N_2_, 0/−78 °C, and 6 h; (ii) 1.0 equiv. of 4‐formyl‐2‐methoxyphenyl trifluoromethanesulfonate (FMT), 0.05 equiv. of SmI_2_, 50 °C and 12 h; 1.0 equiv. of 4‐formyl‐2‐methoxyphenyl trifluoromethanesulfonate (VBPT), 2.2 equiv. of SmI_2_, 7.0 equiv. of triethylamine, 10.5 equiv. of H_2_O, RT and 24 h. b) Synthesis, closed‐loop recyclability and end‐of‐life disposal routes of bio‐based polyurethane networks. The dissociation temperature can be significantly adjusted (≈70–120 °C) by modifying the *R*‐substituents, allowing it to suit a wide range of application scenarios.

In this study, we develop a fully bio‐based polyurethane system composed of renewable bisphenols containing degradable ester bonds, glycerol as a crosslinking agent, and lysine‐derived diisocyanate (LDI)^[^ ^40^ ^]^ (**Scheme** **1**). The bisphenols are synthesized via a streamlined coupling reaction based on the Tishchenko reaction, which disproportionates two aldehydes into an ester,^[^ ^41^, ^42^, ^43^ ^]^ achieving 100% atom utilization of bio‐based benzaldehydes (e.g., *p*‐hydroxybenzaldehyde, vanillin, and syringaldehyde^[^ ^36^ ^]^) without introducing petroleum‐based segments. These bisphenols serve as key building blocks for a covalent adaptable network (CAN) polyurethane with excellent reprocessability and thermal responsiveness. The resulting crosslinked polyurethane exhibits reconfigurable shape memory behavior, with a glass transition temperature (*T*_g_) of 28–54 °C, allowing deformation at room temperature and shape recovery upon heating. At higher temperatures (≈140 °C), dynamic bond exchange enables topological reconfiguration, ensuring recyclability and tunable performance. This fully bio‐based design enhances environmental sustainability by providing an eco‐friendly disposal pathway while offering potential applications in smart packaging, adaptive materials, and reusable devices.

## 2 Results and Discussion

### 2.1 Synthesis and Characterization of Bio‐Based Bisphenols

As illustrated in Scheme 1a, bio‐based renewable bisphenols were successfully synthesized through an innovative and efficient method. This approach involved two key steps: first, phenolic hydroxyl groups were protected using triflic anhydride (Tf_2_O); second, the Tishchenko coupling reaction was employed to connect bio‐based monomers, followed by the deprotection of phenolic hydroxyl groups. The products of the protection and coupling reactions were confirmed through ^1^H NMR spectroscopy (Figures S1–S6, Supporting Information). The highlight of this method lies in the dual functionality of SmI_2_, which simultaneously catalyzes the Tishchenko coupling reaction and facilitates the selective deprotection of phenolic hydroxyl groups. This enables the entire process to be performed as a “one‐pot” reaction, significantly reducing the number of reaction steps and aligning with the principles of green chemistry.

Initial attempts at synthesis (Table S1, Supporting Information) faced challenges due to the reactivity of phenolic hydroxyl groups, which hindered the direct application of the Tishchenko reaction to bio‐based benzaldehydes. Strategies using benzyl and methoxy protecting groups with NaH as the coupling catalyst failed to achieve effective deprotection. Similarly, triflate (TfO) protection combined with various catalysts, including NaH, SmI_2_, Ni(cod)_2_, and RuH_2_(PPh_3_)_4_, showed limited success, with only RuH_2_(PPh_3_)_4_ and SmI_2_ exhibiting catalytic activity.

Optimization identified SmI_2_ as the ideal catalyst, capable of selectively deprotecting phenolic hydroxyl groups while preserving ester bonds. This enabled a “one‐pot” synthesis combining coupling and deprotection. After the coupling reaction, bio‐based bisphenols were obtained directly by adding SmI_2_, triethylamine, and water, eliminating the need for further purification and simplifying the process.

This streamlined method significantly reduces energy consumption and reagent waste while simplifying the production process, providing an efficient and sustainable approach to synthesizing renewable bisphenols. The incorporation of naturally degradable ester bonds ensures an eco‐friendly end‐of‐life disposal route, addressing the non‐degradable nature of BPA and mitigating its associated environmental concerns.

The final products—vanillin‐based bisphenol (VBP), *p*‐hydroxybenzaldehyde‐based bisphenol (*p*‐HBP), and syringaldehyde‐based bisphenol (SBP)—were characterized by ^1^H NMR spectroscopy (Figures S7–S9, Supporting Information). These renewable bisphenols serve as sustainable alternatives to BPA, combining excellent functional properties with a reduced environmental footprint, making them promising candidates for replacing petrochemical‐derived compounds.

### 2.2 Preparation and Characterization of Bio‐Based Renewable Polyurethane Films and PBA‐Polyurethane Film

As illustrated in Scheme 1b, bio‐based renewable polyurethane (PU) films were synthesized using bio‐based bisphenols, lysine‐derived diisocyanate (LDI), and glycerol in specific proportions. All monomers used in the synthesis are bio‐based: LDI is derived from *L*‐lysine,^[^ ^40^ ^]^ glycerol originates from natural oils,^[^ ^44^ ^]^ and the self‐designed bio‐based bisphenols incorporate ester bonds, enabling the resulting PUs to be degradable. The PU films were denoted as XPU‐*p*, where X represents the bio‐based bisphenol type (V for VBP, H for *p*‐HBP, and S for SBP), and *p* indicates the molar percentage of hydroxyl groups from glycerol in the PUs to the total ones. Similarly, APU‐20 based on petroleum‐based BPA was synthesized for performance comparison. As a trifunctional monomer, glycerol acts as a crosslinking agent, with its content modulating the degree of crosslinking in the polymer network, thereby influencing the thermal and mechanical properties of the films. Additionally, the electronic effects of substituents on the phenyl rings of various bio‐based bisphenols impact the phenol‐carbamate interactions, enabling the customization of covalent adaptable networks (CANs) and the thermal and mechanical properties of PU films.

The polymerization process was confirmed by Fourier Transform Infrared Spectroscopy (FTIR). Figure S10 (Supporting Information) displays the FTIR spectra of VBP, LDI, and VPU‐*p*. The absence of an absorption band near 2260 cm⁻¹ confirms the complete reaction of isocyanate groups during polymerization. Key spectral features include aliphatic C‐H stretching vibrations at 2867 and 2935 cm^−^¹ (CH_2_ and CH_3_ groups), C═O stretching peaks at 1739 cm^−^¹ (from LDI), 1710 cm^−^¹ (from bio‐based bisphenols), and 1703 cm^−^¹ (from carbamates), along with characteristic N‐H vibrations at 3358 and 1539 cm^−^¹. These findings confirm the successful synthesis of bio‐based PU films. Additionally, PU films synthesized using *p*‐HBP and SBP (HPU‐20 and SPU‐20) were also prepared, enabling an investigation into the effects of varying bisphenol substituents on material properties. Their FTIR spectra are provided in Figure S11 (Supporting Information). The FTIR spectrum of APU‐20 is provided in Figure S12 (Supporting Information).

The crosslinked structures of the PU films were evaluated using gel fraction analysis via tetrahydrofuran (THF) extraction at 25 °C for 24 h. All bio‐based PU vitrimer films exhibited gel fractions exceeding 90.0 wt.% (Table S2 and Figure S13, Supporting Information), indicating a highly crosslinked network with effective functional group conversion. For instance, in VPU‐20, the gel fraction increased from 89.2 to 94.3 wt.% with higher glycerol content, reflecting an increase in crosslinking density and a more compact polymer network.

### 2.3 Dynamic Dissociation Properties of Linear Model Compounds

The bio‐based polyurethanes synthesized in this study belong to covalent adaptable networks and incorporate dynamic covalent bonds, specifically phenol‐carbamate bonds, which confer temperature‐responsive behavior. At low temperatures, these polyurethanes predominantly contain stable carbamate bonds. However, at elevated temperatures, the carbamate bonds theoretically dissociate, releasing phenolic hydroxyl and isocyanate groups. To investigate this dissociation process, we aimed to confirm the re‐liberation of phenolic hydroxyl and isocyanate groups at high temperatures.

**Figure 1:**
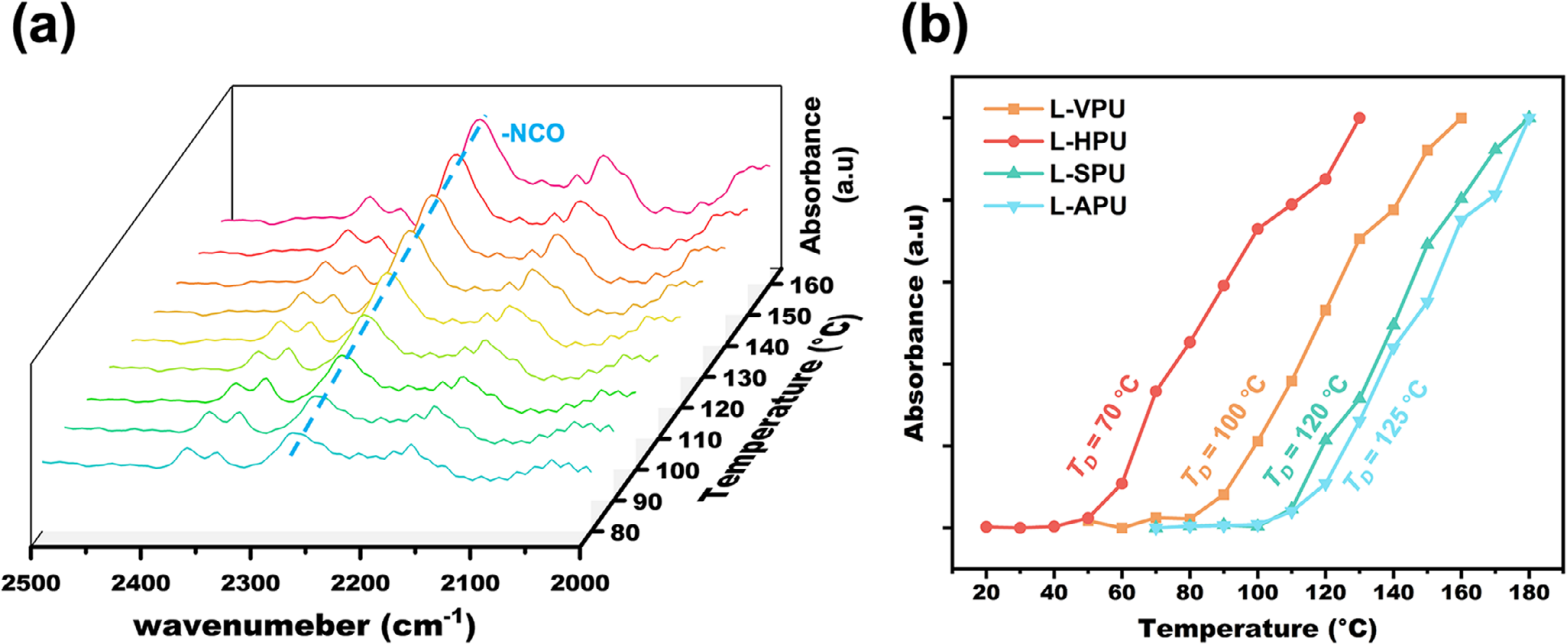
Temperature‐dependent in situ ATR‐IR spectra illustrating the dissociation of −NCO groups in linear model compounds: a) L‐VPU and b) a comparison of normalized free −NCO peak intensities (≈2270 cm^−1^) for L‐VPU, L‐HPU, L‐SPU, and L‐APU. The term *T*_D_ refers to the dynamic covalent bond dissociation temperature.

Given that crosslinked polyurethane networks are unsuitable for in situ attenuated total reflection infrared (in situ ATR‐IR) detection due to their crosslinked nature, the crosslinker glycerol was replaced with 1‐decanol to synthesize small‐molecule linear analogues (as shown in Figure S14, Supporting Information). The initial dissociation temperatures of these small‐molecule analogues, a critical characteristic of the dynamic covalent networks, were determined from temperature‐dependent in situ ATR‐IR spectra. **Figure** **1**a illustrates the spectra for the VBP‐based linear analogue (L‐VPU) at varying temperatures. Notably, at ≈100 °C, a distinct increase in the isocyanate peak at 2270 cm^−^¹ was observed, indicating significant dissociation of the phenol‐carbamate bond and the release of isocyanate groups. Similar dissociation behavior was detected in linear analogues derived from *p*‐HBP, SBP and BPA, as shown in Figure S15 (Supporting Information).

The dissociation temperatures exhibited a clear dependence on the electronic effects of substituents on the benzene ring of the bio‐based bisphenols (Figure 1b). The *p*‐HBP system, without methoxy substitutions, displayed the lowest dissociation temperature (≈70 °C), while the SBP system, with two methoxy substitutions, showed the highest dissociation temperature (≈120 °C). This trend suggests that the electron‐donating methoxy groups on the benzene ring increase the stability of the phenol‐carbamate bond, requiring higher temperatures for dissociation. These findings provide valuable insights into the tunable thermal responsiveness of bio‐based polyurethanes, demonstrating the influence of benzene ring substituents on the dissociation behavior of dynamic covalent bonds.

The distinction in dynamic exchange activity between the linear analogs derived from petroleum‐based BPA and those based on the bio‐based system was investigated. As shown in Figure S15a (Supporting Information), the dissociation temperature observed via in situ ATR‐IR (≈120–130 °C) for the bio‐based system is comparable to that of the SBP‐based analog, indicating its potential as a substitute for BPA in dynamic properties. This behavior is attributed to the combined effects of the electron‐withdrawing ester bond and the electron‐donating methoxy groups in SBP, which collectively produce a similar impact on the phenol‐carbamate bonds as the ‐C(CH_3_)_2_‐ in BPA. Furthermore, it is demonstrated that the bio‐based polyurethane exhibits a broad range of adjustable thermal responsiveness (≈70–120 °C) and a lower dissociation temperature compared to the BPA‐based system.

### 2.4 Dynamic Exchange Properties of Small Molecule Model Compounds

Many dynamic covalent bonds can undergo bond exchange due to dynamic equilibrium, even within temperature ranges where dissociation is minimal. In Section 2.3, it was demonstrated that these bonds dissociate to release isocyanate groups at elevated temperatures. To further explore their dynamic exchange nature at lower temperatures, the bond exchange behavior of VBP‐based phenol‐carbamate bonds was investigated at 75 °C.

Although significant dissociation of phenol‐carbamate bonds was observed only at ≈100 °C in in situ ATR‐IR studies, it is confirmed that dynamic exchange occurs at 75 °C in a model reaction. As illustrated in Figure S16a (Supporting Information) Steps **1, 2** and **3**, VBP was first reacted with cyclohexyl isocyanate (CHI) to form the VBP‐CHI adduct. Benzylamine (BA) was then introduced into the system and reacted at 75 °C for 24 h. Due to the higher reactivity of amines toward isocyanate groups compared to phenolic hydroxyl groups, the dissociated isocyanate groups preferentially reacted with BA, forming irreversible urea bonds (**c** and **d** in Step **3** of Figure S16a,b, Supporting Information).

In addition, nuclear magnetic resonance (NMR) analysis confirmed the reappearance of VBP (**a** and **e** in Step 3 of Figure S16a,b, Supporting Information), indicating that the original carbamate bonds in the VBP‐CHI adduct underwent a dynamic exchange process, exchanging to urea bonds and regenerating VBP. This demonstrates the robust dynamic behavior of phenol‐carbamate bonds, even at moderate temperatures. To align more closely with the behavior of phenolic compounds in the actual polymer system, small phenolic molecules were used in place of BA for further verification. As shown in Figure S16a (Supporting Information) Steps **1**, **2**, and **4**, the phenol‐carbamate bonds exhibited similar dynamic exchange behavior, successfully completing bond exchanging under the same conditions (Figure S16c, Supporting Information). These findings confirm that the dynamic covalent phenol‐carbamate bonds possess excellent exchangeability, enabling bond reorganization at moderate temperatures, which is critical for their application in adaptable polymer systems.

### 2.5 Thermal and Mechanical Properties

Materials with varying glass transition temperatures (*T*_g_) are suitable for diverse applications. For crosslinked polyurethane networks, *T*_g_ is primarily influenced by the degree of cross‐linking and the structure of the polymer backbone. Differential scanning calorimetry (DSC) was used to compare a series of polyurethanes, including VPU‐*p* with varying cross‐linker contents, XPU‐20 synthesized from different bio‐based bisphenols and APU‐20 from BPA.

**Figure 2:**
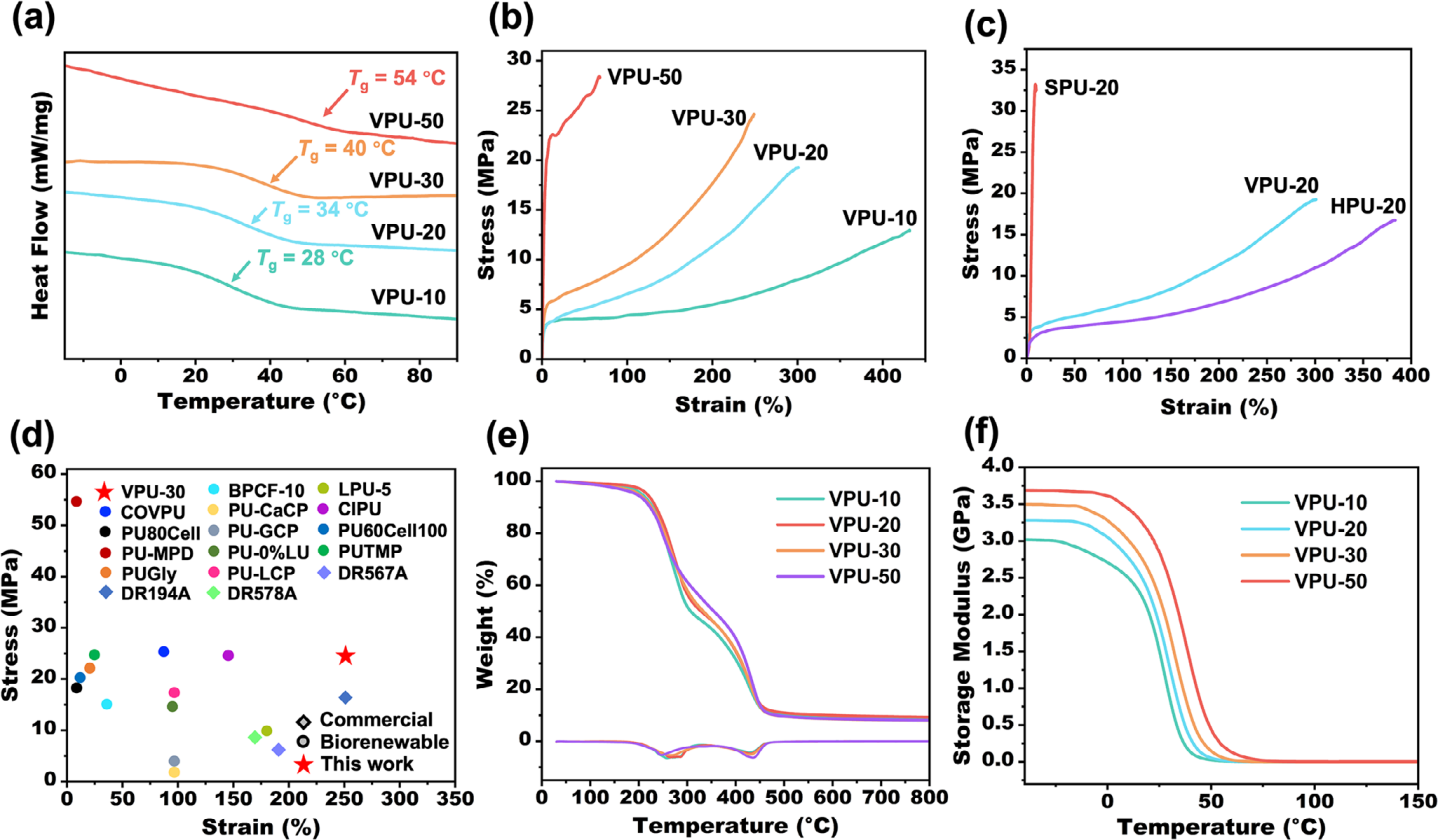
a) DSC curves of VPU‐*p*. b) The stress–strain curves of VPU‐*p*. c) The stress–strain curves of VPU‐20, HPU‐20 and SPU‐20. d) Mechanical performance of VPU‐30 in this work compared with some biorenewable polyurethanes reported previously and commercial nonrenewable polyurethanes.^[^ ^45^, ^46^, ^47^, ^48^, ^49^, ^50^, ^51^, ^52^, ^53^, ^54^, ^55^ ^]^ e) TGA and DTG curves of VPU‐*p*; heating rate: 10 °C min^−1^; atmosphere: N_2_. f) Storage modulus curves of VPU‐*p*.

As shown in **Figure** **2**a, the *T*_g_ values of polyurethanes derived from VBP increased from 28 °C for VPU‐10 to 54 °C for VPU‐50 with higher glycerol content, indicating that *T*_g_ is strongly affected by the cross‐linking degree. However, the *T*_g_ values for VPU‐20, HPU‐20, and SPU‐20—produced from three different bisphenols—showed minimal variation, suggesting that minor methoxy substituents on the benzene ring have negligible impact on *T*_g_ when the polymer backbone remains consistent (Figure S17a, Supporting Information). The *T*_g_ of APU‐20 was measured to be 60 °C, significantly higher than that of bio‐based polyurethanes with the same crosslinking degree. This difference is likely due to the absence of ester bonds in BPA. Furthermore, APU‐20 was found to exhibit pronounced brittleness, rendering it unsuitable for mechanical property testing.

It is noteworthy that an endothermic melting peak was observed near 143 °C in the DSC curve of SPU‐20, which is attributed to partial crystallization (Figure S17b, Supporting Information). This is likely due to the symmetric methoxy substituents on SBP promoting regular polymer arrangement and forming hydrogen bonds with amino groups, further enhancing crystallization. Overall, the tunable *T*_g_ range (28 to 54 °C) demonstrates the versatility of fully bio‐based polyurethane vitrimers across various applications.

The mechanical properties of the polyurethanes were evaluated through tensile test (Figure 2b). The VPU series exhibited excellent mechanical performance, with properties dependent on the cross‐linking degree. VPU‐50, with the highest cross‐linking degree (50% glycerol hydroxyl content), demonstrated a maximum tensile strength of 28.6 ± 2.1 MPa and an elongation at break of 70 ± 3.9%. VPU‐10, with the lowest cross‐linking degree, exhibited a tensile strength of 13.1 ± 1.5 MPa and a remarkable elongation at break of 430 ± 15.2%. Moderate cross‐linking degrees, as seen in VPU‐20 and VPU‐30, achieved a balance of toughness and strength, with elongations at break of 250–300% and tensile strength of 20–25 MPa. The mechanical properties of HPU‐20 and SPU‐20 were also tested (Figure 2c). VPU‐30, with its well‐balanced performance, was compared to polyurethanes reported in the literature and commercial counterparts.^[^ ^45^, ^46^, ^47^, ^48^, ^49^, ^50^, ^51^, ^52^, ^53^, ^54^, ^55^ ^]^ The bio‐based polyurethane demonstrates exceptional mechanical properties, including high tensile strength, excellent elongation at break, and remarkable toughness (**Table** **1** and Figure 2d), highlighting its superior competitiveness. Moreover, most of the bio‐based PUs exhibited excellent toughness, with energy absorption at fracture reaching ≈30 MJ m^−^^3^ (Table 1), surpassing many previously reported polyurethane materials.^[^ ^45^, ^46^, ^47^, ^48^, ^49^, ^50^, ^51^, ^52^, ^53^, ^54^, ^55^ ^]^ This behavior could be attributed to the enhanced molecular interactions within the polymer network, including hydrogen bonding between urethane groups and the electronic effects of methoxy substituents, which may influence chain mobility and network flexibility, ultimately contributing to the improved toughness.

**Table 1:** Mechanical and thermal properties of bio‐based PU networks.

Sample	Strength [MPa]	Elongation at break [%]	Tensile modulus [MPa]	Storage Modulus [GPa]	Toughness [MJ m^−3^]	*T*_g_ [°C]
VPU‐10	13.1 ± 1.5	431 ± 15.2	203 ± 24	3.0	29.1 ± 2.1	28
VPU‐20	19.6 ± 1.2	302 ± 12.8	228 ± 18	3.3	29.0 ± 1.8	34
VPU‐30	24.7 ± 1.8	249 ± 13.3	293 ± 28	3.5	30.5 ± 2.3	40
VPU‐50	28.6 ± 2.1	69.2 ± 3.9	644 ± 48	3.7	16.1 ± 1.2	50
HPU‐20	16.7 ± 1.6	385 ± 16.4	108 ± 15	2.0	29.2 ± 2.4	34
SPU‐20	33.2 ± 2.3	10.0 ± 1.6	774 ± 83	/	1.6 ± 0.2	35

Thermal stability, another critical property of polyurethane vitrimers, was analyzed using thermogravimetric analysis (TGA) under a nitrogen atmosphere. The temperature corresponding to 5% mass loss (*T*_d5%_) was ≈220–230 °C for the VPU series, indicating good thermal stability (Figure 2e). The derivative thermogravimetric (DTG) curves revealed two distinct mass loss stages. The first stage, occurring between 250 and 300 °C, corresponds to the cleavage of carbamate bonds and the release of LDI fragments.^[^ ^56^ ^]^ The second stage, between 400 and 450 °C, corresponds to the decomposition of ester bonds.^[^ ^57^ ^]^ The TGA results for HPU‐20 and SPU‐20 were consistent with those of the VPU series (Figure S18, Supporting Information). Variations in the molecular weights of SBP, VBP, and HBP resulted in differences in the proportions of mass loss during the second stage. Due to the higher molecular weight of SBP, SPU‐20 exhibited a larger proportion of mass loss in the second stage compared to VPU‐20 and HPU‐20. These results highlight the thermal stability, mechanical performance, and tunable properties of bio‐based polyurethanes, demonstrating their potential for diverse high‐performance applications.

DMA tests were performed on VPU‐*p* and HPU‐20 to evaluate their thermomechanical properties (Figure 2f; Figure S19, Supporting Information). The storage modulus (*E*') of all bio‐based polyurethanes exceeded 2 GPa in the glassy state. Notably, VPU‐50 exhibited a storage modulus as high as 3.7 GPa (Figure 2f and Table 1), surpassing that of several commonly used commercial thermosetting epoxy resins, such as DER331 and DEN431.^[^ ^58^ ^]^ This superior *E*' of bio‐based polyurethane is attributed to the stiffness provided by the crosslinking network and the aromatic structure of the bio‐based bisphenol. Since the SPU‐20 and APU‐20 films are rather brittle, the DMA test was not carried out.

### 2.6 Thermal Processing Recyclable Properties

In industrial applications, waste thermoplastic polymers are commonly recycled through thermomechanical processes, such as extrusion and injection molding.^[^ ^59^ ^]^ However, traditional thermosetting plastics cannot be recycled by these methods due to their permanent cross‐linked network structures. In contrast, covalent adaptable networks with dynamic covalent bonds enable bond dissociation and recombination at elevated temperatures, allowing thermosetting polymers to be reprocessed and remolded. This approach combines the advantages of thermosetting materials, such as high rigidity, solvent resistance, and dimensional stability, with the reprocessability typically associated with thermoplastics.

**Figure 3:**
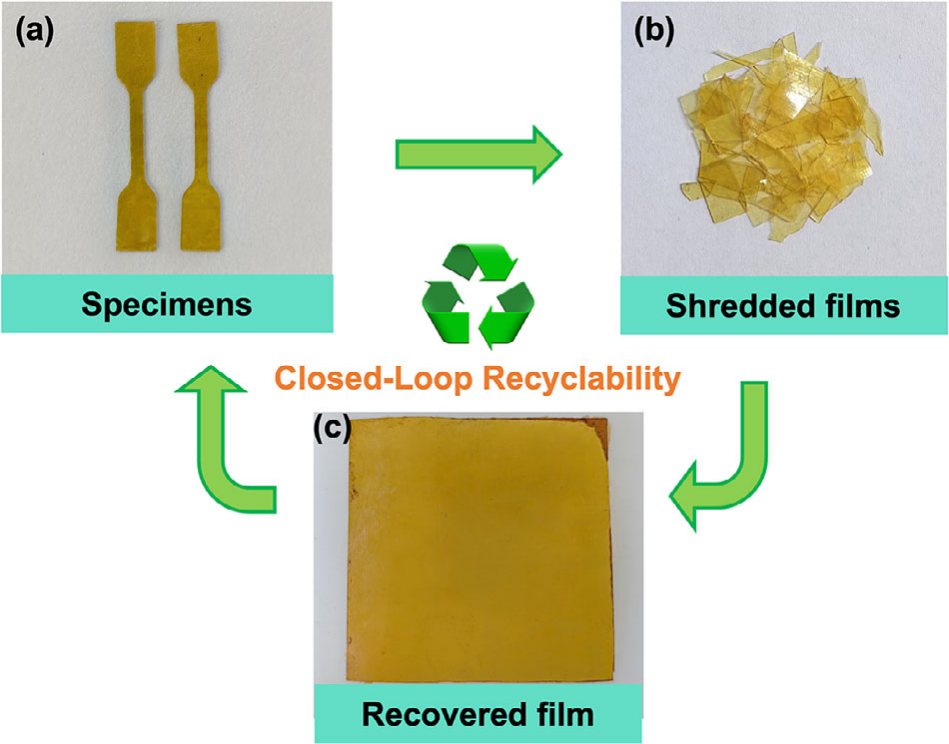
Thermomechanical recycling of VPU‐20 was performed by hot pressing at 150 °C under 3 MPa for 8 min: a) specimens prepared from the original film, b) shredded VPU‐20 films derived from the specimens, and c) the intact film obtained after hot pressing.

**Figure 4:**
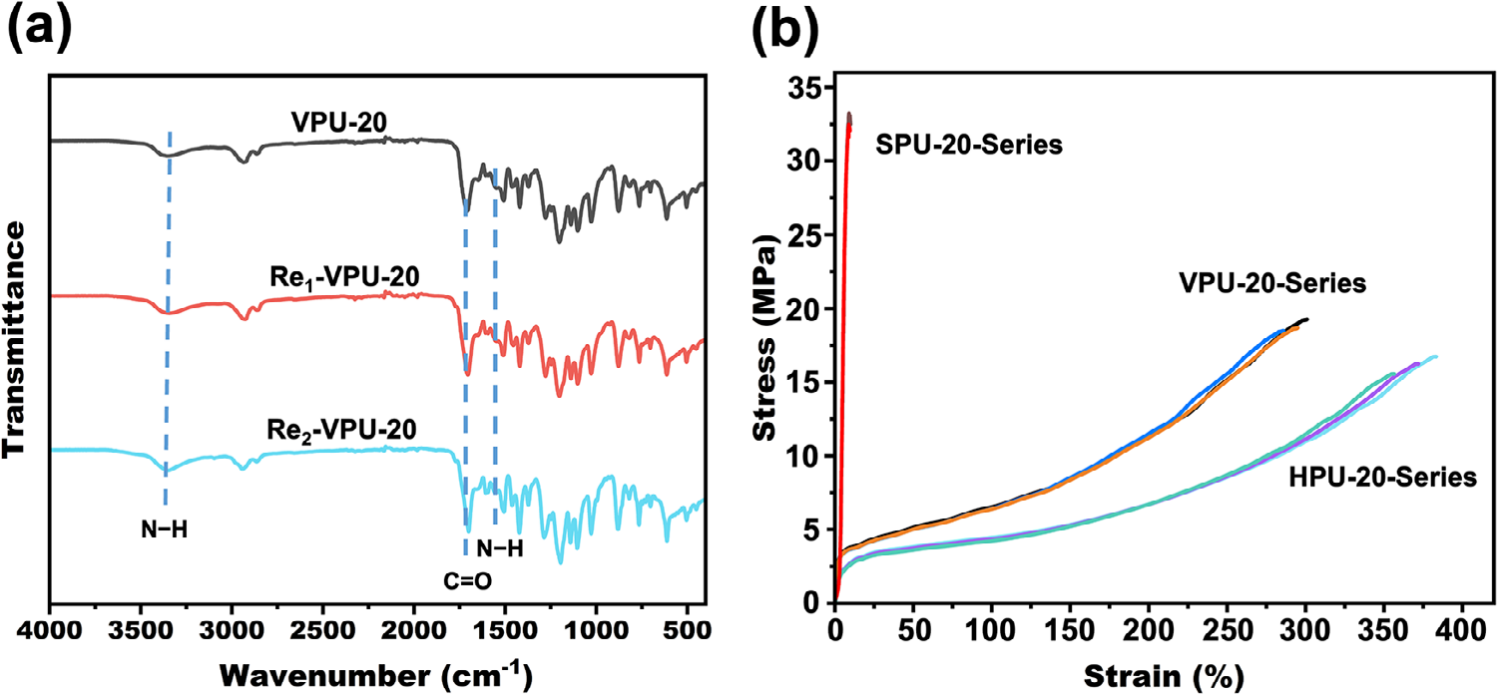
Comparison of FTIR spectra and mechanical properties before and after reprocessing: a) FTIR spectra of the original and reprocessed VPU‐20, and b) representative tensile stress–strain curves of the different XPU‐20 series, which include the original samples and samples reprocessed once and twice.

The recyclability of the VPU‐20 material was simulated through a thermomechanical reprocessing process. As shown in **Figure** **3**, shredded VPU‐20 films were subjected to hot pressing at 150 °C under 3 MPa for 8 min, resulting in the restoration of intact films. The chemical structure of VPU‐20 after two thermomechanical reprocessing cycles was characterized by FTIR spectroscopy (**Figure** **4**a). The characteristic peaks of VPU‐20 remained unchanged after two cycles, confirming that its chemical structure was preserved during reprocessing.

The mechanical properties of VPU‐20, HPU‐20, and SPU‐20 after thermomechanical reprocessing were also evaluated (Figure 4b). The VPU‐20′s tensile strength and elongation at break after one cycle retained 95.4% and 96.8% of the original values, respectively, and after two cycles, these values remained at 93.3% and 95.3%. These results demonstrate that the thermomechanical method effectively achieves closed‐loop recyclability of VPU vitrimer materials without significant degradation in their mechanical properties or chemical structure.

Recyclability tests were also performed on the other two bio‐based renewable HPU‐20 and SPU‐20. As shown in Figure S20 (Supporting Information), the FTIR spectra of HPU and SPU after two cycles of thermomechanical reprocessing exhibited no significant changes, indicating the retention of their chemical structures. Similarly, tensile tests were conducted on them, and the results are summarized in **Table** **2**. These results confirm that HPU and SPU also exhibit excellent recyclability, comparable to VPU‐20.

**Table 2:** Mechanical properties of the original and reprocessed bio‐based PU vitrimers using hot processing.

Sample^a)^	Strength [MPa]	Elongation at break [%]	Tensile modulus [MPa]
VPU‐20	19.6 ± 1.2	301 ± 12.8	228 ± 18
Re_1_‐VPU‐20	18.7 ± 2.2	292 ± 13.2	233 ± 21
Re_2_‐VPU‐20	18.3 ± 1.9	286 ± 11.4	237 ± 24
HPU‐20	16.7 ± 1.6	384 ± 16.4	108 ± 15
Re_1_‐HPU‐20	16.2 ± 2.4	371 ± 14.7	114 ± 17
Re_2_‐HPU‐20	15.4 ± 2.1	356 ± 15.3	117 ± 13
SPU‐20	33.2 ± 2.3	10.0 ± 1.6	774 ± 83
Re_1_‐SPU‐20	31.3 ± 2.8	9.5 ± 2.1	782 ± 72
Re_2_‐SPU‐20	29.5 ± 1.6	9.1 ± 1.8	779 ± 79

^a)^ The numerical subscript represents the number of reprocessing cycles.

The findings highlight the potential of bio‐based polyurethanes containing dynamic covalent bonds to achieve closed‐loop recyclability, combining the superior performance of thermosetting materials with the sustainable advantage of reprocessability.

### 2.7 Shape Memory and Cyclic Properties

Shape memory polymer materials (SMPs) can adopt a predefined shape, temporarily fix a deformed state under external stimuli, and recover their original shape upon environmental changes.^[^ ^60^, ^61^ ^]^ Temperature‐induced SMPs, the most common type, usually rely on transitions between the glassy and rubbery states. When heated above the glass transition temperature (*T*_g_), the molecular chains changes from a “frozen” to an “unfrozen” state, enabling shape recovery through entropy elasticity. Typically, SMPs consist of a fixed phase, often formed by cross‐linking points, which preserves the initial shape, and a reversible phase that responds to temperature changes.

For conventional SMPs, the initial state is determined by the fixed‐phase topology during material curing and cannot be altered post‐curing. In contrast, the polyurethane (PU) material developed in this study leverages the temperature‐responsive behavior of covalent adaptable networks.^[^ ^62^, ^63^, ^64^ ^]^ Through dynamic covalent bond exchange at elevated temperatures, the topology (initially fixed phase) of the PU material can be reconfigured, enabling reshaping and broadening its application for shape‐memory functionality.

**Figure 5:**
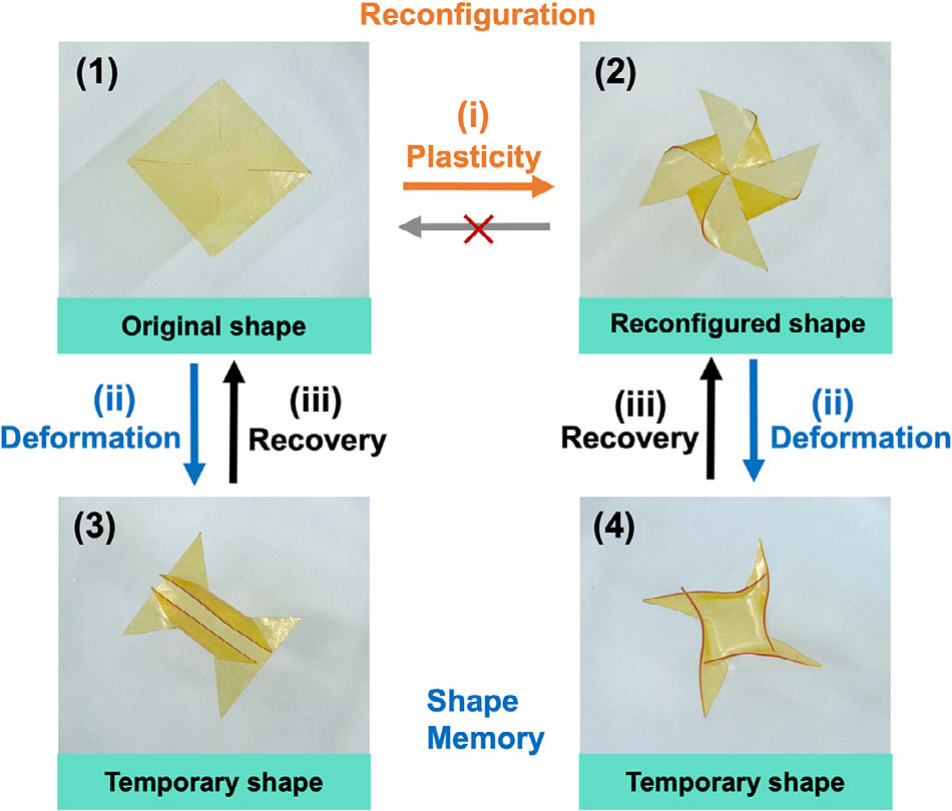
A wide variety of shapes can be made by utilizing the shape memory and shape reconfiguration. (**1**) Planar shape—original shape. (**2**) Windmill shape reconfigured from **1**. (**3**) Temporary bridge shape from **1**. (**4**) Temporary flower shape from **2**.(i) 140 °C for 20 min; (ii) Heat to 70 °C; (iii) Cool to 25 °C.

To demonstrate the reconfigurable shape memory capability, VPU‐20 was utilized to create various models (**Figure** **5**). Starting with the original planar shape (**1**), the model was deformed into a temporary bridge shape (**3**) by heating to 70 °C (above *T*_g_) and cooling to room temperature to fix the shape. Upon reheating to 70 °C, the model fully recovered to the original planar shape, showcasing excellent shape memory ability. Additionally, the planar shape (**1**) was deformed into a windmill shape (**2**) and reconfigured by heating at 140 °C for 20 min, resulting in a reconfigured PU windmill. As demonstrated, the reconfigured windmill shape (**2**) could be further deformed into a temporary flower shape and subsequently recovered to its initial windmill shape upon reheating. These results provide a clear and intuitive demonstration of the shape memory performance and topological reconfigurability of the fully bio‐based PU films.

Moreover, the shape memory performance of VPU‐20 was also demonstrated via the single‐axis tensile shape memory experiment (Figure S21a–f, Supporting Information). Shape memory cycle experiments and recovery ratios for original and reconfigured specimens are summarized in Figure S22 and Table S3 (Supporting Information). The average recovery ratio over three cycles was 96.5%, confirming the excellent shape memory performance of VPU‐20. The reconfigurability of VPU‐20′s topology was then evaluated through dynamic covalent bond exchange at evaluated temperatures (Figure S21c–f, Supporting Information). The reconfiguratied shape was also verified through shape memory cycles with an average recovery ratio of 95.8%, demonstrating retained shape memory performance after reconfiguration.

These results highlight the dual capabilities of VPU‐20, combining traditional shape memory functionality with the reconfigurability enabled by dynamic covalent networks, significantly broadening its potential applications in adaptive and reusable polymer systems.

### 2.8 Degradation Properties

The majority of polyurethane (PU) is derived from non‐renewable petroleum‐based resources, with its end‐of‐life typically involving either incineration (≈45%), which contributes to greenhouse gas emissions and exacerbates global warming, or disposal in landfills (≈50%), causing soil and ecosystem pollution.^[^ ^2^, ^65^ ^]^ To address these environmental challenges, sustainable materials must not only originate from bio‐based, renewable resources but also demonstrate full‐cycle degradability, producing pollution‐free byproducts.^[^ ^66^ ^]^ Even if discarded directly into the natural environment, such materials should not contribute to “white pollution”. Therefore, polymers are preferably designed to be degradable while ensuring that their degradation products do not impose environmental burdens. Most commercially available degradable polymers, such as polylactic acid (PLA), achieve natural degradability due to their ester bonds. In this study, ester bonds were introduced during the modification of bio‐based monomers. Since the polymers are entirely derived from bio‐based monomers, their degradation products are environmentally benign. The natural degradability of ester bonds, as widely validated in PLA materials, is further confirmed by their accelerated degradation in alkaline environments.

The degradability of bio‐based bisphenol monomers was evaluated using ^1^H NMR in an alkaline environment. As shown in Figure S23 (Supporting Information), the vanillin‐based bisphenol (VBP) monomer degraded completely in a 0.1 m NaOD solution (DMSO‐*d*₆/D₂O = 1:1, v/v), with the ester bond peak at 5.33 ppm disappearing. Similarly, *p*‐HBP and SBP monomers also exhibited complete degradation of their ester bonds, as indicated by the disappearance of peaks at 5.35 and 5.34 ppm, respectively (Figures S24 and S25, Supporting Information). These results confirm that bio‐based bisphenol monomers possess excellent degradation performance under alkaline conditions.

**Figure 6:**
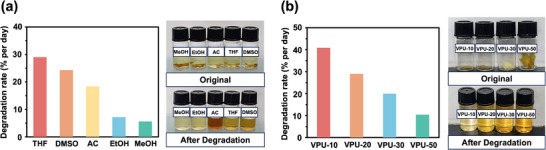
a) The bar chart of the degradation rate of VPU‐20 in 0.1 m NaOH solutions (organic solvent/H₂O = 1:1, v/v) with different solvents (THF, DMSO, AC, EtOH, MeOH). b) The bar chart of the degradation rate of VPU‐*p* in 0.1 m NaOH solutions (THF/H₂O = 1:1, v/v).

The degradation behavior of bio‐based polyurethanes was then studied by immersing the polymers in 0.1 m NaOH solutions with various solvent mixtures. VPU‐20 was selected as the representative material, and its degradation rates were determined in solvent mixtures of THF, dimethyl sulfoxide (DMSO), acetone (AC), ethanol (EtOH), methanol (MeOH), and H₂O (organic solvent/H₂O = 1:1, v/v). As shown in **Figure** **6**a, the degradation rates followed a decreasing trend from THF (≈29% per day) to MeOH (≈5.6% per day). The faster degradation in THF can be attributed to its higher affinity for the polymer, enhancing solubility and swelling, which facilitates greater contact between the polymer chains and NaOH. In contrast, the lower solubility of the polymer in MeOH results in reduced solvent diffusion and slower degradation.

The influence of cross‐linking density on degradation rates was further investigated. As shown in Figure 6b, VPU‐10 exhibited the fastest degradation rate (≈41% per day), while VPU‐50 degraded at the slowest rate (≈10% per day). The increased cross‐linking density in VPU‐50 creates a tighter polymer network, limiting solvent diffusion and prolonging degradation time. Conversely, materials with lower cross‐linking densities, such as VPU‐10, allow for greater solvent permeation and diffusion, accelerating degradation. During polymer degradation, material volume expansion was observed, likely due to the reaction of NaOH with ester bonds on the surface and within the polymer, resulting in alkaline hydrolysis.

The degradation performance of HPU‐20 and SPU‐20 was evaluated in a 0.1 m NaOH solution mixed with THF and compared to that of VPU‐20 (Figure S26, Supporting Information). Similar trends were observed across the bio‐based polyurethanes, with degradation rates influenced by the solvent's affinity. For comparison, petroleum‐based BPA polyurethane was tested, showing only mild degradation, likely attributed to the breakdown of urethane bonds (Figure S26, Supporting Information). However, its degradation rate was very low (≈1.7% per day), suggesting that it is unlikely to degrade in a natural environment lacking alkalinity. These findings highlight the consistent degradability of bio‐based polyurethane materials under alkaline conditions, reinforcing their potential as sustainable, closed‐loop alternatives.

## 3 Conclusion

In summary, we have developed an SmI₂‐mediated strategy that integrates Tishchenko coupling with one‐pot phenol deprotection, enabling an efficient synthesis of bio‐based bisphenols with degradable ester bonds. This approach is broadly applicable to bio‐derived aldehydes and phenolic hydroxyl‐containing compounds, offering a sustainable alternative to petroleum‐based BPA and addressing key environmental challenges in polyurethane production. The resulting bio‐based bisphenols serve as a versatile platform for high‐performance, closed‐loop polyurethanes that combine renewable sourcing, recyclability, and rapid degradability. By incorporating phenol‐carbamate dynamic covalent bonds, these polyurethanes exhibit excellent reprocessability while retaining mechanical integrity across multiple recycling cycles. Furthermore, the dissociation temperatures of these bonds can be precisely tuned (≈70–120 °C) by modifying the electronic properties of benzaldehyde precursors, allowing for tailored thermal response. With outstanding shape memory performance (recovery efficiency >96.5%), these materials offer programmable functionality and structural adaptability. This work demonstrates a scalable, green approach to bio‐based polyurethane vitrimers, highlighting the potential of renewable aromatic building blocks for designing high‐performance, recyclable, and degradable materials.

## Conflict of Interest

The authors declare no conflict of interest.

## Supporting information



Supporting Information

## Data Availability

The data that support the findings of this study are available from the corresponding author upon reasonable request.
