# Supplementary material for: Hydrolyzable Bio‐Based Bisphenols Enabled by the Tishchenko Reaction for Polyurethane Vitrimers with Closed‐Loop Recyclability
Source: Adv Sci (Weinh). 2025 Apr 11;12(26):2503152. doi: 10.1002/advs.202503152 (PMC12245132; doi:10.1002/advs.202503152)
Supplement: Supplementary file 1 — Supporting Information [file ADVS-12-2503152-s001.docx]

**Supporting Information**

**Hydrolyzable** **Bio-Based Bisphenols Enabled by the Tishchenko Reaction for Polyurethane Vitrimers with Closed-Loop Recyclability**

*Jiewen Wang^1^, Hongru Qiang^1^, Rong Huang^1^, Dan Zhao^1^, Zihan Tong^1^, Zhen Fan^1,^*, Jianzhong Du^2,3,4,^* and Yunqing Zhu^1,^**

*^1^Department of Polymeric Materials, School of Materials Science and Engineering, Tongji University, Shanghai 201804, China*

*^2^**Department of Gynaecology and Obstetrics, Shanghai Key Laboratory of Anesthesiology and Brain Functional Modulation, Clinical Research Center for Anesthesiology and Perioperative Medicine, Translational Research Institute of Brain and Brain-Like Intelligence, Shanghai Fourth People’s Hospital, School of Medicine, Tongji University, Shanghai 200434, China*

*^3^Key Laboratory of Advanced Civil Engineering Materials of Ministry of Education, School of Materials Science and Engineering, Tongji University, 4800 Caoan Road, Shanghai 201804, China*

*^4^School of Materials Science and Engineering, East China University of Science and Technology, Shanghai 200237, China*

**Table of Contents**

1. Materials 5

2. Methods 5

2.1 Synthesis of Bio-based Renewable Bisphenols. 5

2.2 Synthesis of Linear Compounds. 6

2.3 Investigation of Dynamic Properties of Small Molecule Model Compounds. 6

2.4 Synthesis of Degradable Covalent Adaptable Networks Polyurethanes and BPA-Polyurethane. 6

2.5 Thermomechanical Reprocessing Performance of Renewable Polyurethanes. 7

2.6 Shape Memory of Renewable Polyurethanes. 7

2.7 Degradation of Bio-based Renewable Bisphenols and Polyurethanes. 8

2.8 Gel Fraction Test. 8

3. General Characterizations 9

3.1 ^1^H Nuclear Magnetic Resonance (^1^H NMR) 9

3.2 Fourier Transform Infrared Spectroscopy (FTIR) 9

3.3 *In-situ* ATR-IR Spectroscopy 9

3.4 Differential Scanning Calorimetry (DSC) 9

3.5 Thermogravimetric Analysis (TGA) 9

3.6 Tensile Test 9

3.7 Dynamic Mechanical Analysis (DMA) 10

4. Figures and Tables 11

4.1 Synthesis of Bio-based Renewable Bisphenols. 11

Table S1. Summary of bio-based bisphenol synthesis experiments with successes and failures 11

Figure S1. ^1^H NMR spectra of 4-formyl-2-methoxyphenyl trifluoromethanesulfonate (FMT) in CDCl_3_. 12

Figure S2. ^1^H NMR spectra of 4-formylphenyl trifluoromethanesulfonate (FT) in DMSO-*d*_6_. 12

Figure S3. ^1^H NMR spectra of 4-formyl-2,6-dimethoxyphenyl trifluoromethanesulfonate (FDT) in DMSO-*d*_6_. 13

Figure S4. ^1^H NMR spectra of vanillin-based bisphenol trifluoromethanesulfonate (VBPT) in DMSO-*d*_6_. 13

Figure S5. ^1^H NMR spectra of *p*-hydroxybenzaldehyde-based bisphenol trifluoromethanesulfonate (*p*-HBPT) in DMSO-*d*_6_. 14

Figure S6. ^1^H NMR spectra of syringaldehyde-based bisphenol trifluoromethanesulfonate (SBPT) in DMSO-*d*_6_. 14

Figure S7. ^1^H NMR spectra of vanillin-based bisphenol (VBP) in DMSO-*d*_6_. 15

Figure S8. ^1^H NMR spectra of *p*-hydroxybenzaldehyde-based bisphenol (*p*-HBP) in DMSO-*d*_6_. 15

Figure S9. ^1^H NMR spectra of syringaldehyde-based bisphenol (SBP) in DMSO-*d*_6_. 16

4.2 FTIR of Bio-based Renewable Polyurethane Films and PBA-Polyurethane Film. 16

Figure S10. The Fourier Transform Infrared spectra (FTIR) of VBP, LDI and VPU-*p*. 16

Figure S11. The Fourier Transform Infrared spectra (FTIR) of VBP, *p*-HBP, SBP, LDI and XPU-20. 17

Figure S12. The Fourier Transform Infrared spectra (FTIR) of BPA, LDI and APU-20. 17

4.3 Gel Fraction Test. 18

Figure S13. Gel fraction test of Bio-based renewable PUs. 18

Table S2. Gel fraction test of Bio-based renewable PUs 18

4.4 Dynamic Dissociation Properties of Linear Model Compounds. 19

Figure S14. Synthesis route of linear compounds for *in-situ* ATR-IR detection. 19

Figure S15. The temperature-dependent *in-situ* ATR-IR spectra of the dissociative −NCO of the linear model compounds : (a)L-APU and (b)L-HPU and (c)L-SPU. 19

4.5 Dynamic Exchange Properties of Small Molecule Model Compounds. 20

Figure S16. The phenol-carbamate dynamic exchange of the small molecular model compound. (a) Reactions of VBP, CHI and BA/Phenol. ^1^H NMR spectra of the stock solution of the (b) step **1**, **2**, **3** and (c) step **1**, **2**, **4** in DMSO-*d*_6_. 20

4.6 Thermal and Mechanical Properties. 20

Figure S17. (a) DSC curves of XPU-20. (b) DSC curves of SPU-20. The third heating curve was analyzed to obtain the *T*_g_ value of all samples. 20

Figure S18. TGA and DTG curves of XPU-20; heating rate: 10 °C/min; atmosphere: N_2_. 21

Figure S19. (a) Tan δ curves of VPU-*p*. (b) Storage modulus and tan δ curves of HPU-20. 21

4.7 Thermal Processing Recyclable Properties. 22

Figure S20. The FTIR spectra of the original and reprocessed (a) HPU-20 and (b) SPU-20. 22

4.8 Shape Memory and Cyclic Properties. 22

Figure S21. The shape memory and shape reconfiguration processes of VPU-20 specimen under the CANs. 22

Figure S22. Shape memory cycle experiments on the stretching of original and reconfigured specimens. Each cycle consists of hot stretching, cooling to fix the shape, and reheating for shape recovery. Detailed experimental procedures are provided in the experimental section. 23

Table S3. Recovery ratio of shape memory cycles for original and reconfiguration specimens. 23

4.9 Degradation Properties. 24

Figure S23. ^1^H NMR spectra of VBP degradation in a 0.1 M NaOD solution (DMSO-*d*₆/D₂O = 1:1, v/v). 24

Figure S24. ^1^H NMR spectra of *p*-HBP degradation in a 0.1 M NaOD solution (DMSO-*d*₆/D₂O = 1:1, v/v). 25

Figure S25. ^1^H NMR spectra of SBP degradation in a 0.1 M NaOD solution (DMSO-*d*₆/D₂O = 1:1, v/v). 25

Figure S26. The bar chart of the degradation rate of XPU-20 (X = V, A, H and S) in 0.1 M NaOH solutions (THF/H₂O = 1:1, v/v). 25

# Materials

Vanillin (99%), syringaldehyde (98%), *p*-hydroxybenzaldehyde (98%), bisphenol A (BPA, 98%), trifluoromethanesulfonic anhydride (Tf₂O, 98%), pyridine (>99%), sodium chloride (NaCl, 99.8%), tetrahydrofuran (THF, 99.9%), cyclohexyl isocyanate (CHI, 98%), 1,4-diazabicyclo[2.2.2]octane (DABCO, 98%), benzylamine (BA, 99%), L-lysine diisocyanate (LDI, 97%), phenol (99%), triethylamine (TEA, 99.5%), dimethyl sulfoxide-𝑑_6_ (DMSO-𝑑_6_, 99.8%), deuterium oxide (D_2_O, 99.9%), and glycerol (99%) were purchased from InnoChem. Sodium deuteroxide (NaOD, 40 wt.% in D_2_O), 1-decanol (98%), and anhydrous magnesium sulfate (MgSO₄, 99.99%) were purchased from Aladdin. Samarium(II) iodide (SmI₂, 0.1 M in THF) was obtained from Macklin. Hydrochloric acid (HCl), dichloromethane (DCM), *n*-hexane (>99%), ethyl acetate (99.8%), *N*,*N*-dimethylformamide (DMF, >99.9%), and acetone (>99%) were purchased from SCRC. Deionized water was prepared in the laboratory. All chemicals were used as received without further purification.

# Methods

## Synthesis of Bio-based Renewable Bisphenols

As illustrated in Scheme 1a, the synthesis of bio-based bisphenols follows a two-step approach. The process begins with the protection of phenolic hydroxyl groups, followed by a ‘one-pot’ method, which includes the Tishchenko coupling reaction and subsequent deprotection. This method is universally applied to all bisphenol preparations in this study, yielding vanillin-based bisphenol (VBP), *p*-hydroxybenzaldehyde-based bisphenol (*p*-HBP), and syringaldehyde-based bisphenol (SBP). Using vanillin-based bisphenol (VBP) as an example, the detailed synthesis steps are described below.

1) Phenolic hydroxyl protection reaction. Vanillin (15.0 g, 98.6 mmol) and pyridine (26.5 g, 335 mmol) were added into a round bottom flask containing dichloromethane (250 mL) and fully stirred to dissolve, and then trifluoromethanesulfonic anhydride (33.6 g, 119 mmol) was slowly added in an ice bath (dry ice and acetone for *p*-hydroxybenzaldehyde) under the protection of nitrogen for 6 h. The reacted solution was washed with 0.5 M HCl, separated, washed with saturated salt water, and finally the organic phase was dried with anhydrous MgSO_4_, then filtered and concentrated under vacuum to obtain a dark brown oily liquid, which was further purified to 24.7 g light yellow oil (4-formyl-2-methoxyphenyl trifluoromethanesulfonate, FMT) by silica gel column chromatography (*n*-hexane : ethyl acetate = 3 : 1). Yield: ~ 88%.

2) ‘one-pot’ method combining the Tishchenko coupling reaction and subsequent deprotection: Tishchenko coupling reaction and deprotection reaction. Under the protection of nitrogen, FMT (8.53 g, 30.0 mmol) and 0.1 M SmI_2_ (15 mL, 1.50 mmol) were added into a round bottom flask, and stirred at 50 °C for 12 h to obtain a yellow suspension containing product (vanillin-based bisphenol trifluoromethanesulfonate, VBPT). According to ^1^H NMR, the yield was ~ 92%. Subsequently, under the protection of nitrogen, 0.1 M of SmI_2_ (330 ml, 33.0 mmol), triethylamine (10.6 g, 105 mmol), and water (2.84 g, 158 mmol) were successively added to the stock solution after the Tishchenko coupling reaction, which was fully stirred and reacted at room temperature for 24 h. The reacted solution was concentrated under vacuum to remove THF, dissolved by adding DCM, washed with 0.5 M HCl, separated, washed with saturated salt water, and finally dried with anhydrous MgSO_4_ and concentrated under vacuum to obtain a dark brown solid. It was further purified to 2.96 g white solid (vanillin-based bisphenol, VBP) by silica gel column chromatography. The eluent used was a mixture of *n*-hexane and ethyl acetate in a 3:1 ratio (v/v). For the p-hydroxybenzaldehyde system, the ratio was adjusted to 9:1 (v/v). Final yield: ~ 65%.

## Synthesis of Linear Compounds

Linear small-molecule analogs were synthesized to facilitate *in-situ* ATR-IR detection of carbamate bond dissociation. General method: VBP (0.76 g, 2.50 mmol), LDI (1.31 g, 5.00 mmol), 1-decanol (0.91 g, 5.00 mmol), THF (2.5 mL), and DABCO (25.3 mg, 1 wt.%) were added to a 10 mL reaction tube and stirred at 50 °C for 2 h. The mixture was placed in a vacuum oven at 45 °C for 24 h to complete the reaction and remove THF. Due to the different reactivities of phenolic and alcoholic hydroxyl groups with LDI, multiple structures may form. Figure S14 illustrates one possible structure.

## Investigation of Dynamic Properties of Small Molecule Model Compounds

Small-molecule model investigations were conducted in two steps, as shown in Figure S15a. 1) Synthesis of VBP-CHI adduct: VBP (12.2 mg, 0.04 mmol), CHI (10.0 mg, 0.08 mmol), and DABCO (0.22 mg) were dissolved in DMSO-*d*_6_ (550 μL) and reacted at 50 °C for 24 h under stirring to form the VBP-CHI adduct; 2) dynamic bond exchange: Benzylamine (BA, 12.9 mg, 0.12 mmol) or an equivalent amount of phenol was added to the stock solution of VBP-CHI adduct, resulting in the formation of 1-benzyl-3-cyclohexylurea and phenyl cyclohexylcarbamate.

## Synthesis of Degradable Covalent Adaptable Networks Polyurethanes and BPA-Polyurethane

Scheme 1b illustrates the synthetic route of the fully bio-based PU vitrimer materials. In this study, this procedure is applicable to all polymer samples based on three degradable bio-based bisphenols—VBP, *p*-HBP, and SBP. Similarly, we synthesized APU-20 using petroleum-based BPA and compared some properties with our PUs.

Taking VPU-20 as an example, the synthesis steps are as follows. Dissolve VBP (584 mg, 1.92 mmol) and glycerol (29.5 mg, 0.32 mmol) in a reaction flask containing 2 mL of THF. Then, add LDI (597 mg, 2.40 mmol) and DABCO (12.1 mg, 1 wt.%) into the reaction flask successively, and heat and stir the mixture at 50 °C for 1 h. After keeping it in an ultrasonic cleaner for 5 min to remove bubbles, transfer the prepolymerized solution to a polytetrafluoroethylene mold and place it in a heating oven at 85 °C for further curing for 12 h. Finally, the material is thermally pressed and cured on a laboratory hot press at 150 °C under 3 MPa for 8 min.

## Thermomechanical Reprocessing Performance of Renewable Polyurethanes

Thermomechanical reprocessing experiments were carried out using a flat curing press. Cut the fully bio-based PU into small pieces with scissors, place it in a metal frame with a certain thickness, cover it with two steel plates covered with two layers of polyimide film (to prevent the film from adhering to the steel plates), and press it at a certain temperature and 3 MPa for 8 min. After cooling to 25 °C, a recyclable fully bio-based PU was obtained. The above operation was repeated twice, that is, the fully bio-based PU materials with two cycles were obtained, and their stress-strain curves and FTIR spectra were tested.

## Shape Memory of Renewable Polyurethanes

(1) Single-Axis Tensile Shape Memory Experiment Steps:

Shape Memory and Shape Reconfiguration: (i) Initial Measurement: record the original length of the tensile specimen as 𝑙_10_. (ii) Stretching and Fixing: heat the specimen to 70 °C (above the material’s 𝑇_𝑔_), stretch it, and fix both ends. Cool it to room temperature, and after 10 min, record the length 𝑙_11_. (iii) Shape Recovery: reheat the specimen to 70 °C, allowing it to shrink freely to a stable length of 𝑙_10_′. (iv) Reconfiguration: fix both ends of the specimen using a weight to achieve a desired length. Place it on a heating plate at approximately 140 °C for 20 min, then cool it to room temperature and record the length 𝑙_13_.

Shape Memory Recovery Ratio:

Record the initial length of the specimen as 𝑙_10_. Simulate the temporary storage process by heating the specimen to 70 °C, then cooling it to room temperature to fix the packaging state. Reheat the specimen to 70 °C, allowing it to unfold naturally and restore its shape. Record the restored length as 𝑙_10_′. The shape memory recovery ratio (𝑅_𝑚_) is calculated using the formula:

R_m_ = 100% − (|*l*_10_ − 𝑙_10_′| / *l*_10_)

Where 𝑅_𝑚_ represents the shape memory recovery ratio.

Additionally, this material demonstrates reconfiguration capability, enabling it to adopt new shapes after initial molding. The shape recovery ratio and other properties can be evaluated using the steps outlined above. The reconfiguration method corresponds to step (iv).

(2) Shape Memory and Reconfiguration of PU Film Models:

The PU film was first cut into squares, then further trimmed diagonally while retaining the center connection, allowing it to be shaped into the windmill shape, bridge shape, and flower shape. For shape memory evaluations, the PU film was heated to 70 °C (above its 𝑇_𝑔_), shaped into the desired form, and cooled to 25 °C to fix the shape. For shape reconfiguration, the film was first deformed into a new shape, then placed on a heating plate at 140 °C for 20 min to set the new shape, and finally cooled to 25 °C to finalize the reconfiguration.

## Degradation of Bio-based Renewable Bisphenols and Polyurethanes

Due to the degradability of bisphenol structure, the degradability of bio-based bisphenol and renewable polyurethane was studied in this experiment. This method is applicable to all bio-based bisphenols and bio-based polyurethanes in this study.

Based-bisphenol small molecule degradation experiment. VBP samples (about 20 mg) were added to 0.1 M NaOD solution (0.55 ml, the volume ratio of DMSO-*d*_6_ and D_2_O was 1/1) and allowed to stand for 24 h at 25 °C.

Degradation experiment of bio-based polyurethane. The VPU-20 sample (about 30 mg) was added to 0.1 M NaOH solution (5 ml, the volume ratio of organic solvent and water was 1/1) at 25 °C for degradation experiments.

The time (*t*) required for almost complete dissolution of the film (residual sample mass < 5%) was monitored. The degradation rate can be calculated according to *w*/*t*, where *w* is generally taken as 95%, representing the sample mass fraction taken when the residual of the sample is less than 5%. In addition, the effect of degradation time of bio-based polyurethane was discussed by changing the type of organic reagent.

## Gel Fraction Test

Gel fraction test was conducted by a solvent immersion method. All sample (∼30 mg) were first dried under vacuum at 70 °C for 24 h and then weighed (*m*_0_). After being immersed in 3 mL of acetonitrile at 25 °C for 24 h, the sample was taken out of the solution. Finally, the sample was dried under vacuum at 70 °C for 24 h and weighed (*m*_1_). The gel fraction of the sample was calculated through 100% × *m*_1_/*m*_0_.

# General Characterizations

## ^1^H Nuclear Magnetic Resonance (^1^H NMR)

^1^H nuclear magnetic resonance（NMR） spectra were recorded using a Bruker AV400 MHz NMR spectrometer at room temperature. Samples (10 mg) were fully dissolved in 0.55 mL of CDCl_3_, DMSO-*d*_6_ or D_2_O, using tetramethyl silane (TMS) as the internal standard.

## Fourier Transform Infrared Spectroscopy (FTIR)

The FTIR spectra of samples were obtained using a Thermo Scientific Nicolet iS20 Fourier Transform Infrared Spectrometer and the transmittance mode was used. The spectra were recorded after 32 scans in a range from 4000 to 400 cm^-1^ with a resolution of 4 cm^-1^.

## *In-situ* ATR-IR spectroscopy

Under a nitrogen atmosphere, different temperatures were provided to the sample using a heating stirrer, and *in-situ* ATR-IR measurements were performed using a Mettler-Toledo ReactIR 702L spectrometer equipped with an MCT detector and a silver halide DiComp probe. For *in-situ* ATR-IR measurement, the resolution is 8 cm^-1^, the number of scans is 32 and the test range is from 650 cm^-1^ to 3000 cm^-1^.

## Differential scanning calorimetry (DSC)

The thermal properties of polymer samples were measured on a TA Instruments Q-2000 DSC. Samples were analyzed in hermetically sealed aluminum pans. The samples were heated from −50 °C to 170 °C at the rate of 10 °C/min under a N_2_ atmosphere and then held for 4 min. Subsequently, the samples were cooled from 170 °C to −50 °C under the same condition. This cycle was repeated for three times.

## Thermogravimetric Analysis (TGA)

The thermal weight loss curves of the bio-based polyurethane films were tested using thermogravimetric analysis (TGA) on TGA 5500 apparatus. The samples were heated from 30 to 1000 °C at a heating rate of 10 °C/min under N_2_ atmosphere. *T*_d5%_ is the temperature corresponding to a 5% loss in sample mass.

## Tensile Test

Tensile tests were conducted on a Universal testing machine (SANS UTM5202) with an extension rate of 10 mm/min. The bio-based polyurethane vitrimer films were cut into dumbbell-shaped sample strips of 35.0 mm in length and 2.0 mm in pitch width using a custom cutter head with the aid of a manual slicer according to standard ISO 527-2-2002. All tests were then carried out according to standard ISO 527–2 at room temperature. Each value was reported as the average of three samples with confidence limits.

## Dynamic Mechanical Analysis (DMA)

The dynamic mechanical properties of the bio-based polyurethane vitrimers were characterized using a TA DMA Q800. The dimensions of the tested samples were 20 mm long, 5 mm wide, and 200 μm thick. The tensile mode was selected and the samples were heated from −40 to 150 °C at a heating rate of 3 °C /min in air with a strain of 0.05% and an oscillation frequency of 1 Hz.

# Figures and Tables

## Synthesis of Bio-based Renewable Bisphenols

### Table S1. Summary of Bio-Based Bisphenol Synthesis Experiments with Successes and Failures

| Sample | Protecting group | Phenolic hydroxyl protection reaction | Tishchenko coupling reaction | Deprotection reaction | Overall result |
| --- | --- | --- | --- | --- | --- |
| Vanillin (ROH) | ROMe | K_2_CO_3_, CH_3_I  ✔︎*^a^* | SmI_2_  ✔︎ | 1. HBr, CH_3_COOH; 2. AlCl_3_   ✖︎ | ✖︎ |
|  |  |  | NaH  ✔︎ |  |  |
|  |  |  | Al(OEt)_3_  ✖︎ | / | ✖︎ |
|  | ROBn | Benzyl bromide  ✔︎ | NaH  ✔︎ | MeOH, Amberlyst-15  ✖︎ | ✖︎ |
|  | ROTBS | Imidazole, tert-Butyldimethylsilyl chloride  ✔︎ | NaH  ✖︎ | / | ✖︎ |
|  | ROTf | Trifluoromethanesulfonic anhydride, pyridine  ✔︎ | NaH  ✖︎ | / | ✖︎ |
|  |  |  | Ni(cod)_2_, IPr  ✖︎ | / | ✖︎ |
|  |  |  | RuH_2_(PPh_3_)_4_  ✔︎ | Et_4_NOH,  1,4-dioxane  ✖︎ | ✖︎ |
|  |  |  | SmI_2_  ✔︎ | Et_4_NOH,  1,4-dioxane  ✖︎ | ✖︎ |
|  |  |  |  | SmI_2_, TEA, H_2_O  ✔︎ | ✔︎ |

*^a^* ‘✔︎’ means the reaction was successful, while ‘✖︎’ means the reaction was not successful.


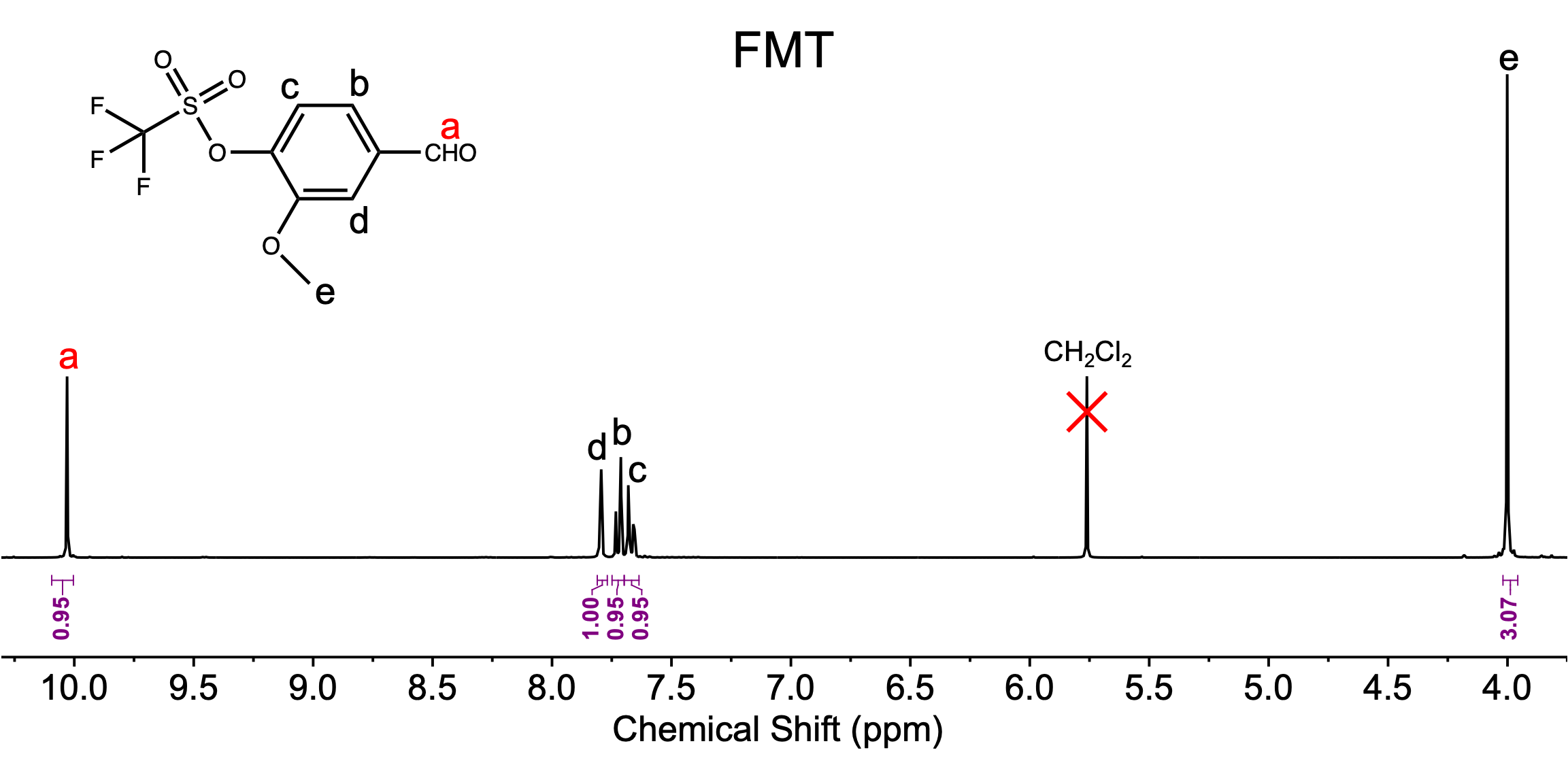


### Figure S1. ^1^H NMR spectrum of 4-formyl-2-methoxyphenyl trifluoromethanesulfonate (FMT) in CDCl_3_.


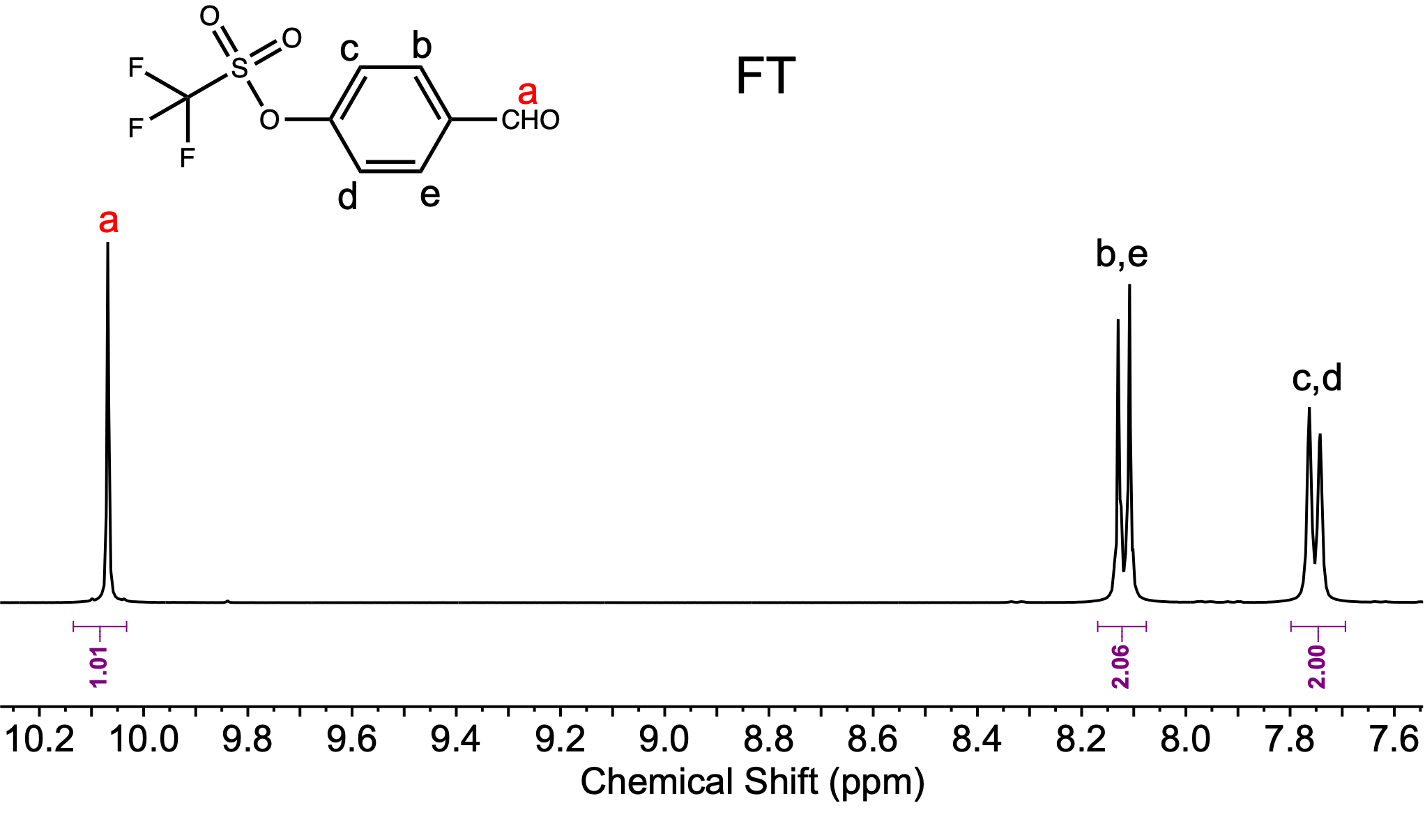


### Figure S2. ^1^H NMR spectrum of 4-formylphenyl trifluoromethanesulfonate (FT) in DMSO-*d*_6_.


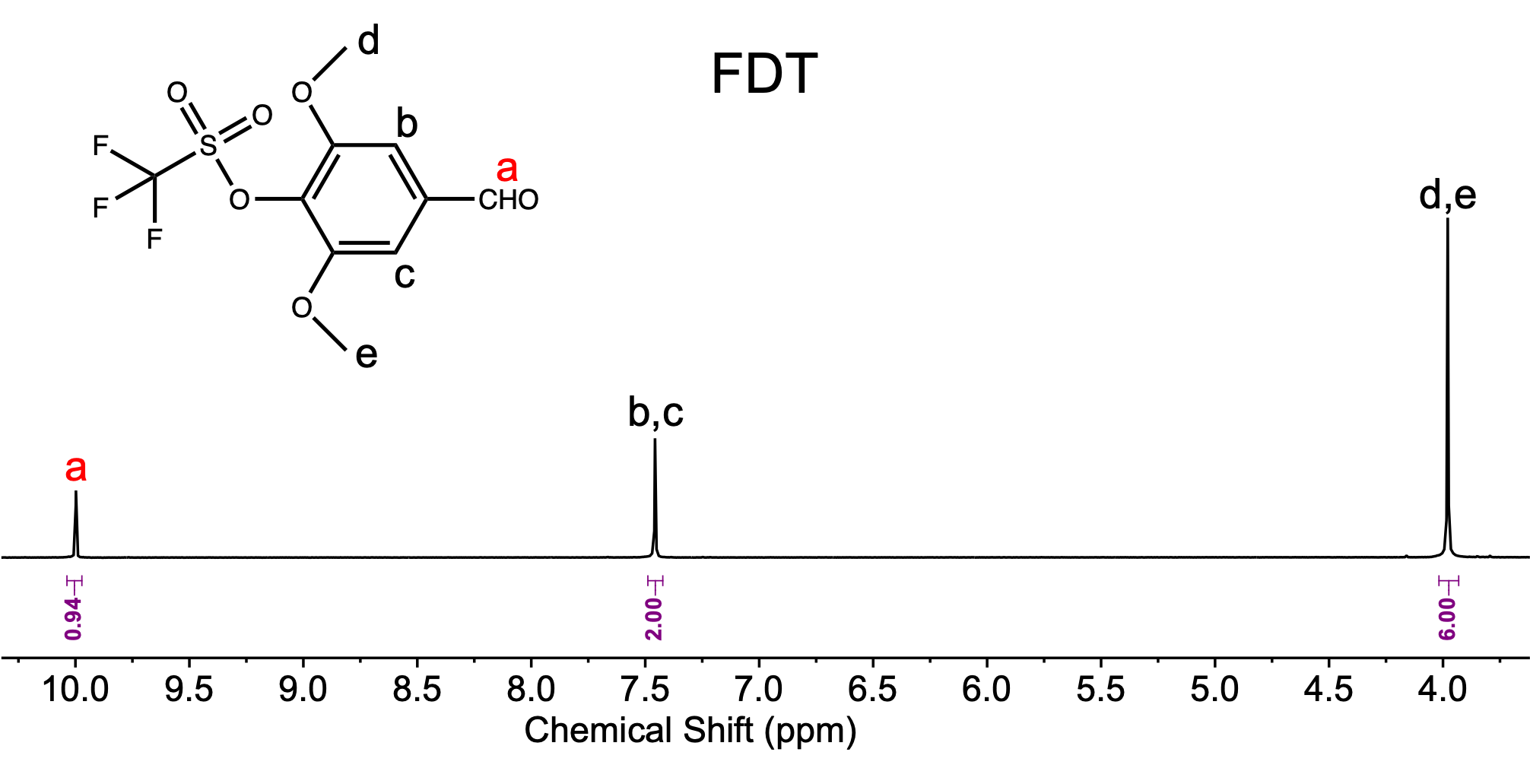


### Figure S3. ^1^H NMR spectrum of 4-formyl-2,6-dimethoxyphenyl trifluoromethanesulfonate (FDT) in DMSO-*d*_6_.


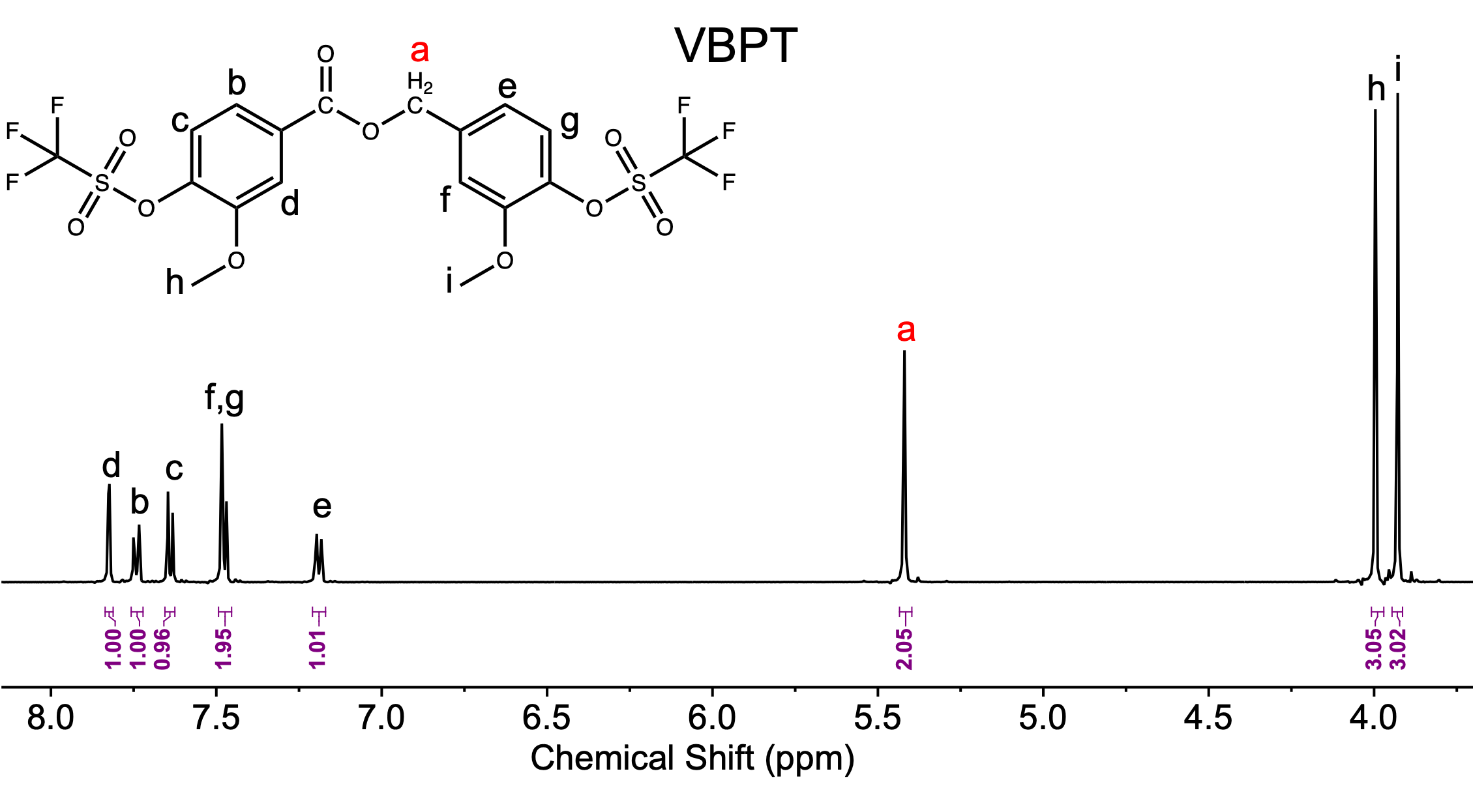


### Figure S4. ^1^H NMR spectrum of vanillin-based bisphenol trifluoromethanesulfonate (VBPT) in DMSO-*d*_6_.


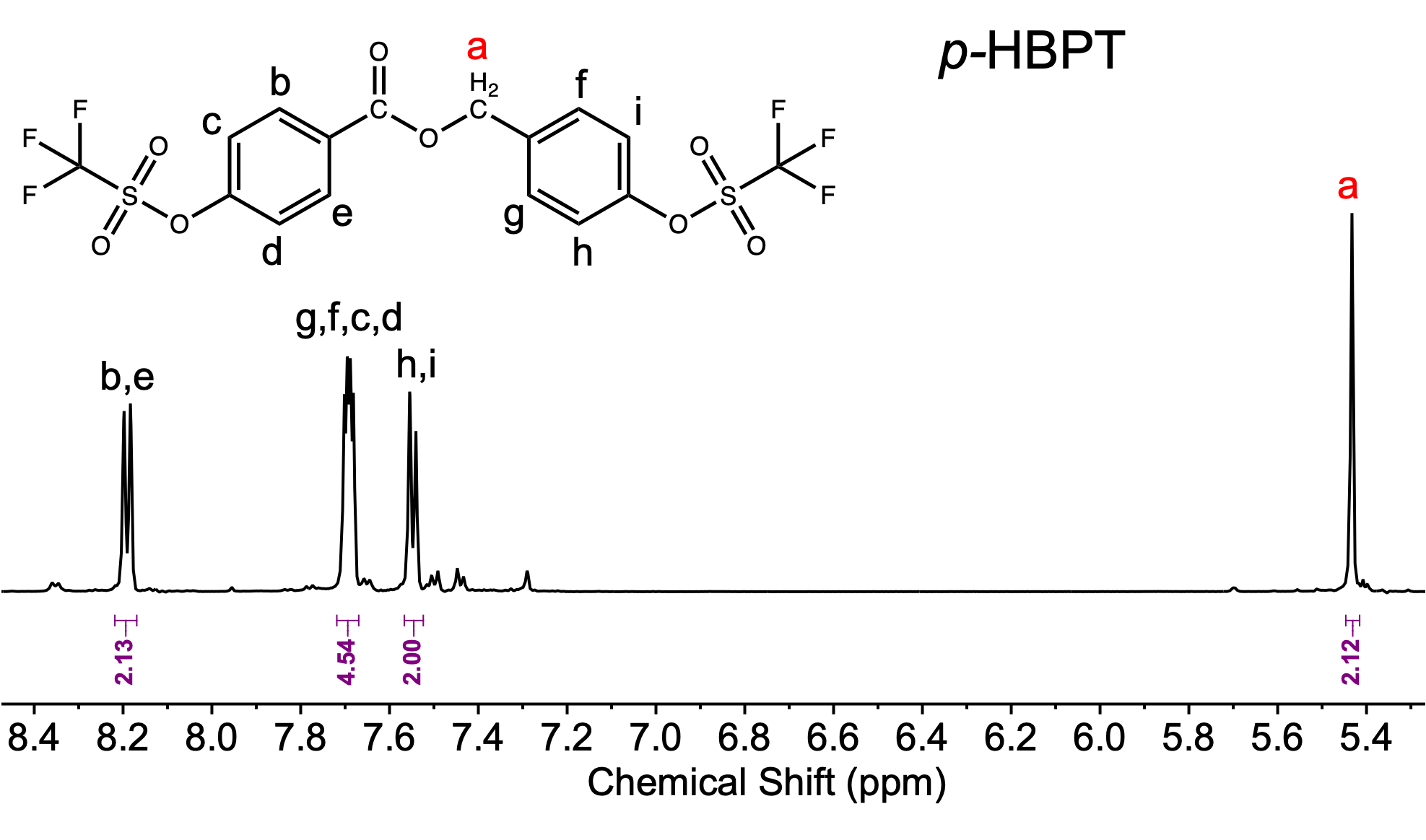


### Figure S5. ^1^H NMR spectrum of *p*-hydroxybenzaldehyde-based bisphenol trifluoromethanesulfonate (*p*-HBPT) in DMSO-*d*_6_.


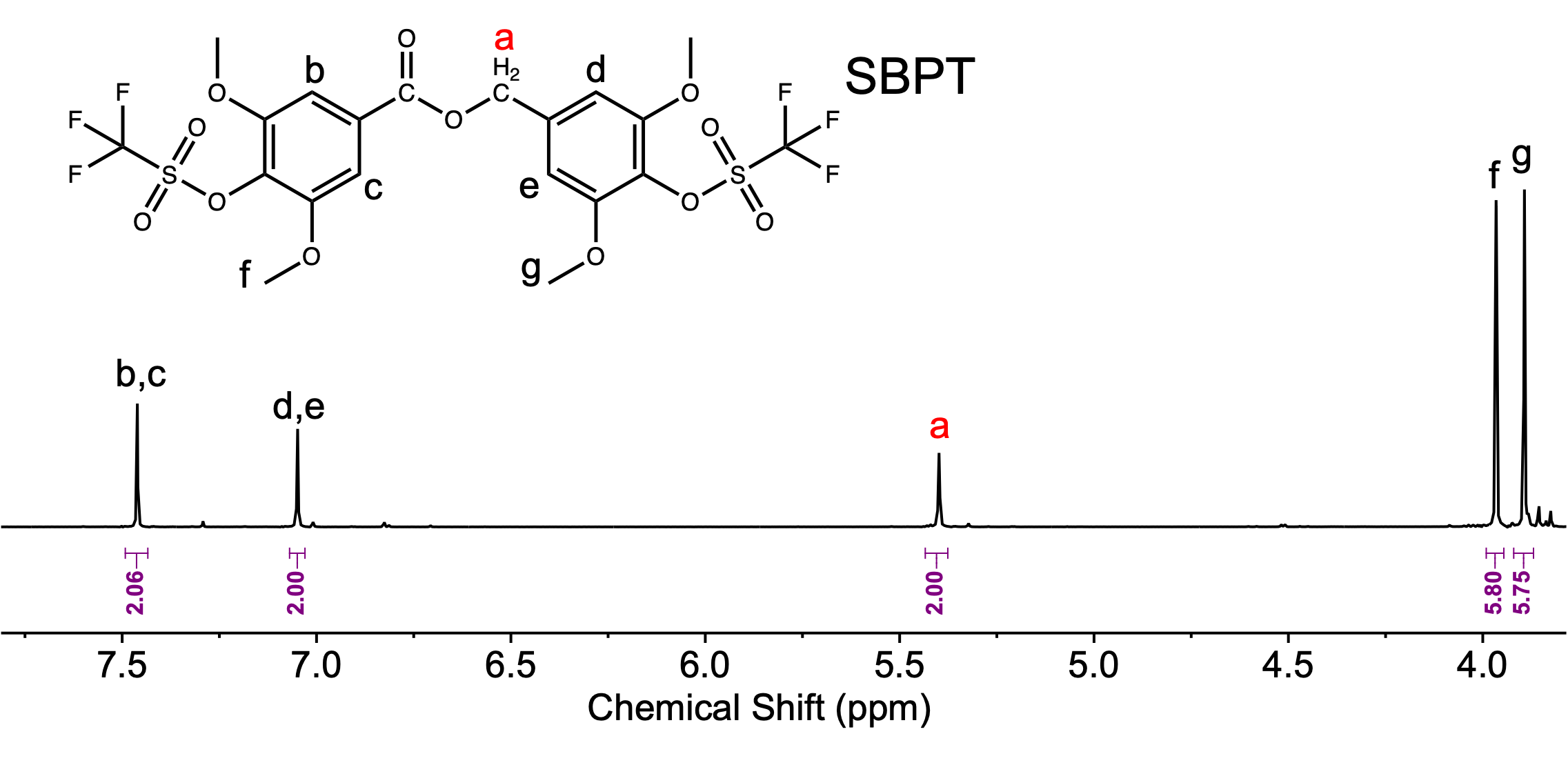


### Figure S6. ^1^H NMR spectrum of syringaldehyde-based bisphenol trifluoromethanesulfonate (SBPT) in DMSO-*d*_6_.


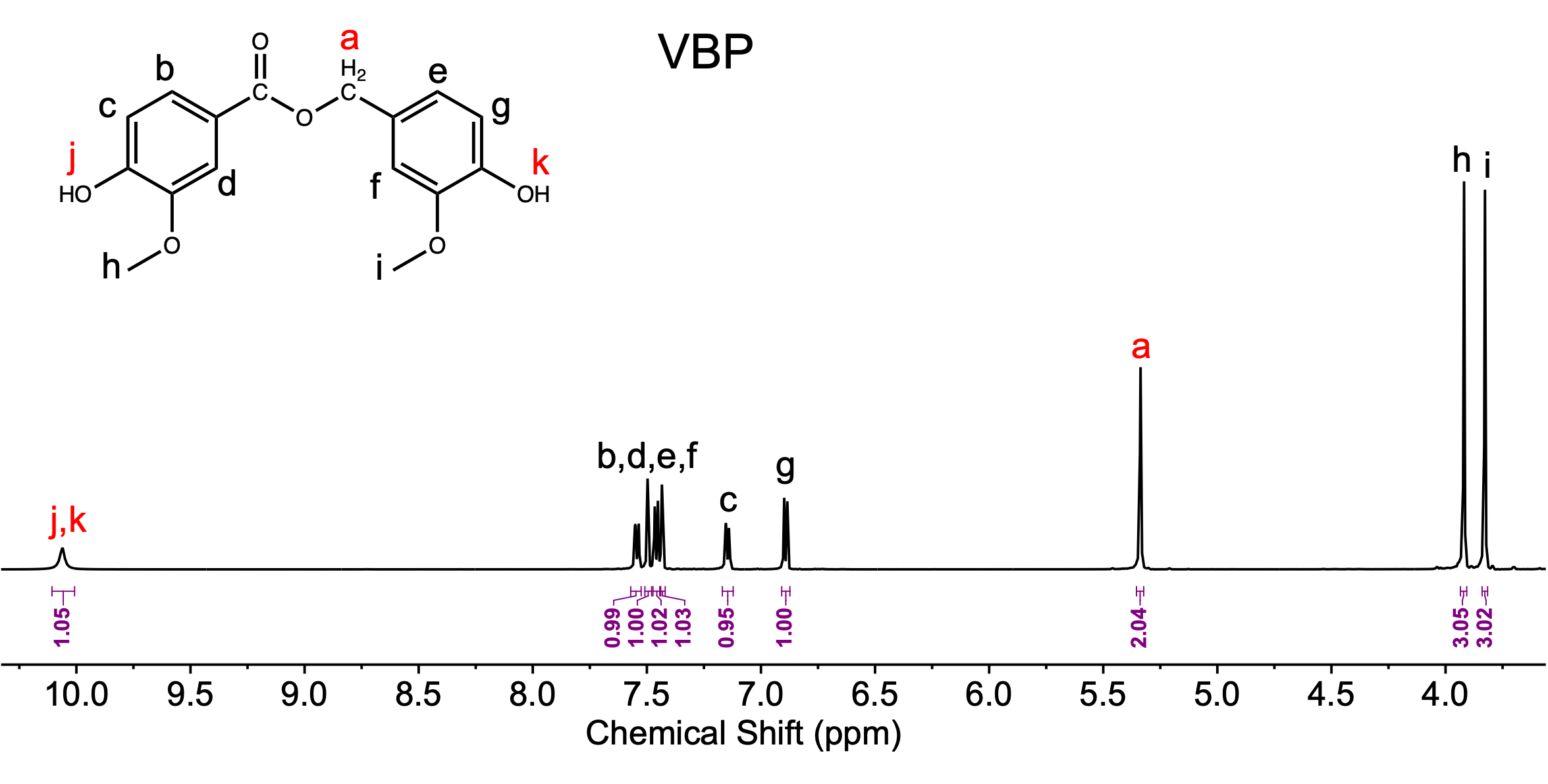


### Figure S7. ^1^H NMR spectrum of vanillin-based bisphenol (VBP) in DMSO-*d*_6_.


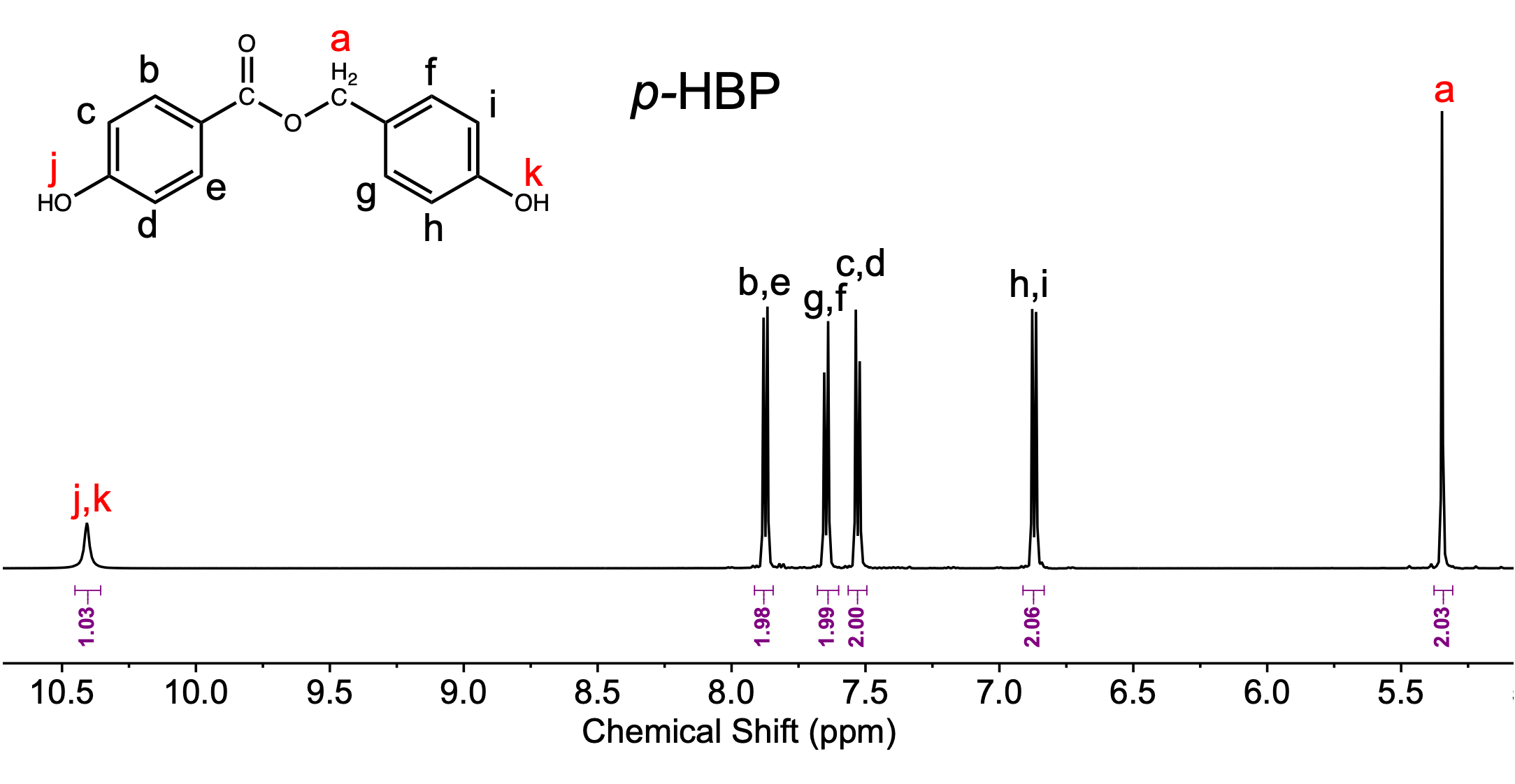


### Figure S8. ^1^H NMR spectrum of *p*-hydroxybenzaldehyde-based bisphenol (*p*-HBP) in DMSO-*d*_6_.


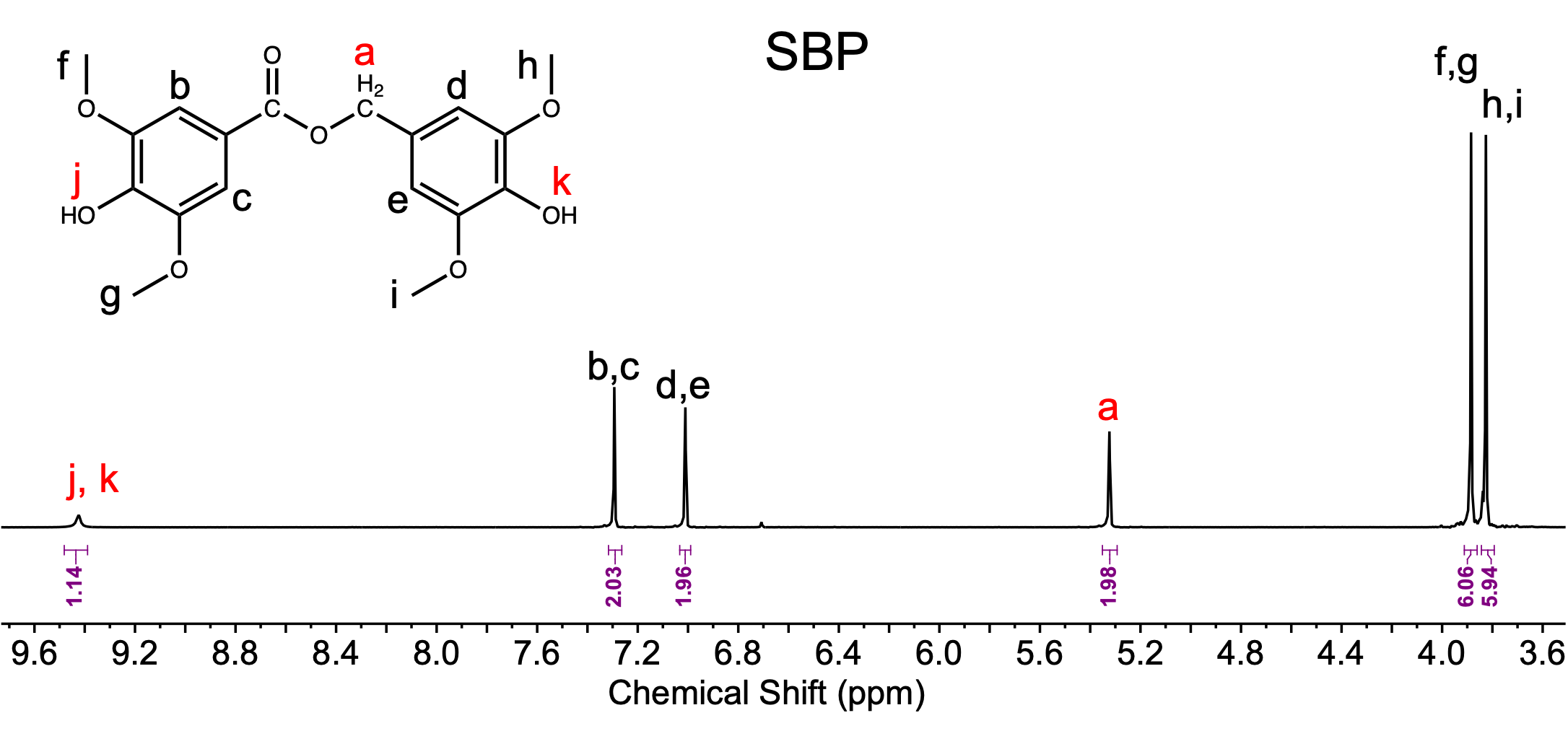


### Figure S9. ^1^H NMR spectrum of syringaldehyde-based bisphenol (SBP) in DMSO-*d*_6_.

## FTIR of Bio-based Renewable Polyurethane Films and PBA-Polyurethane Film


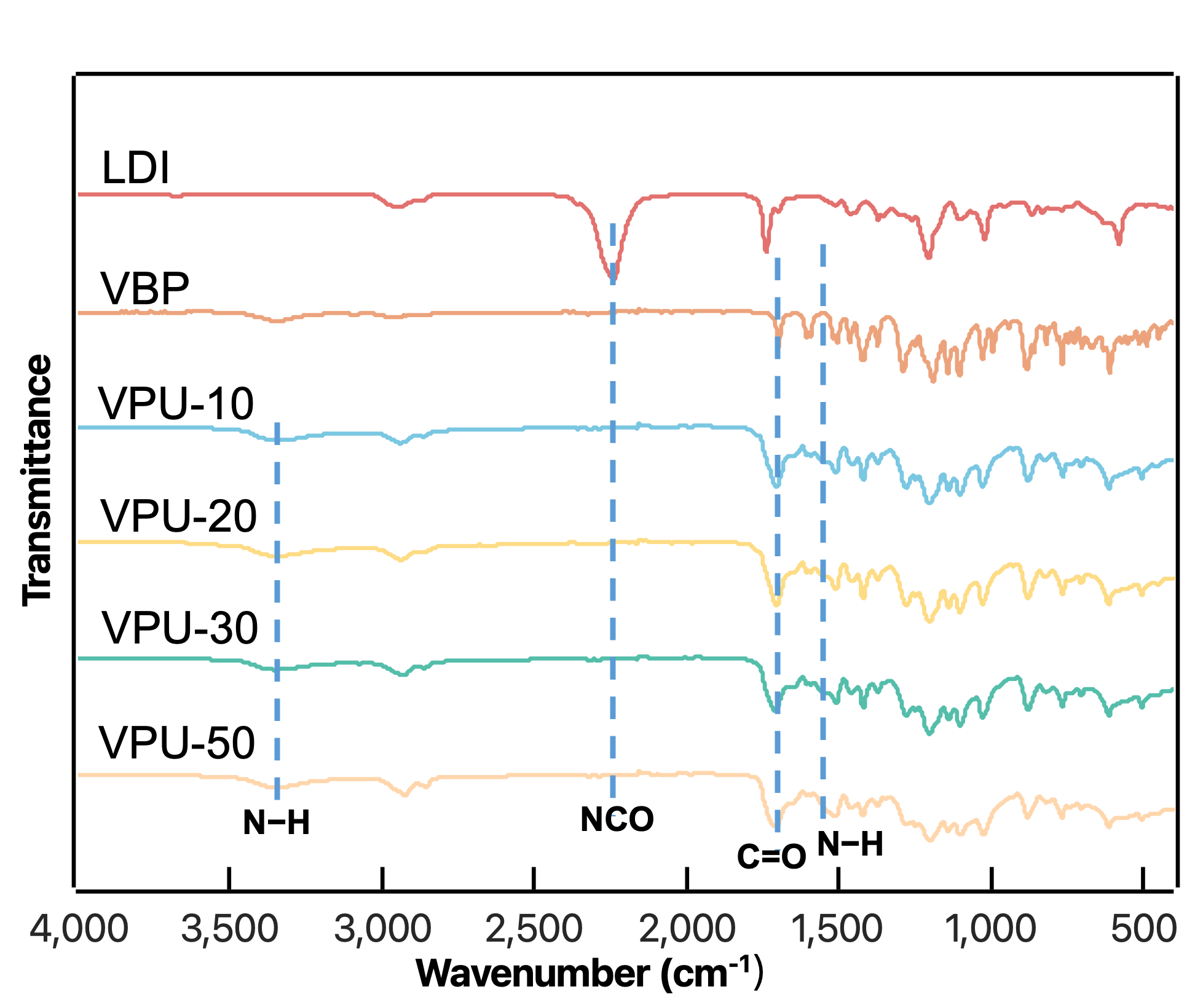


### Figure S10. Fourier Transform Infrared spectra (FTIR) of VBP, LDI and VPU-*p*.


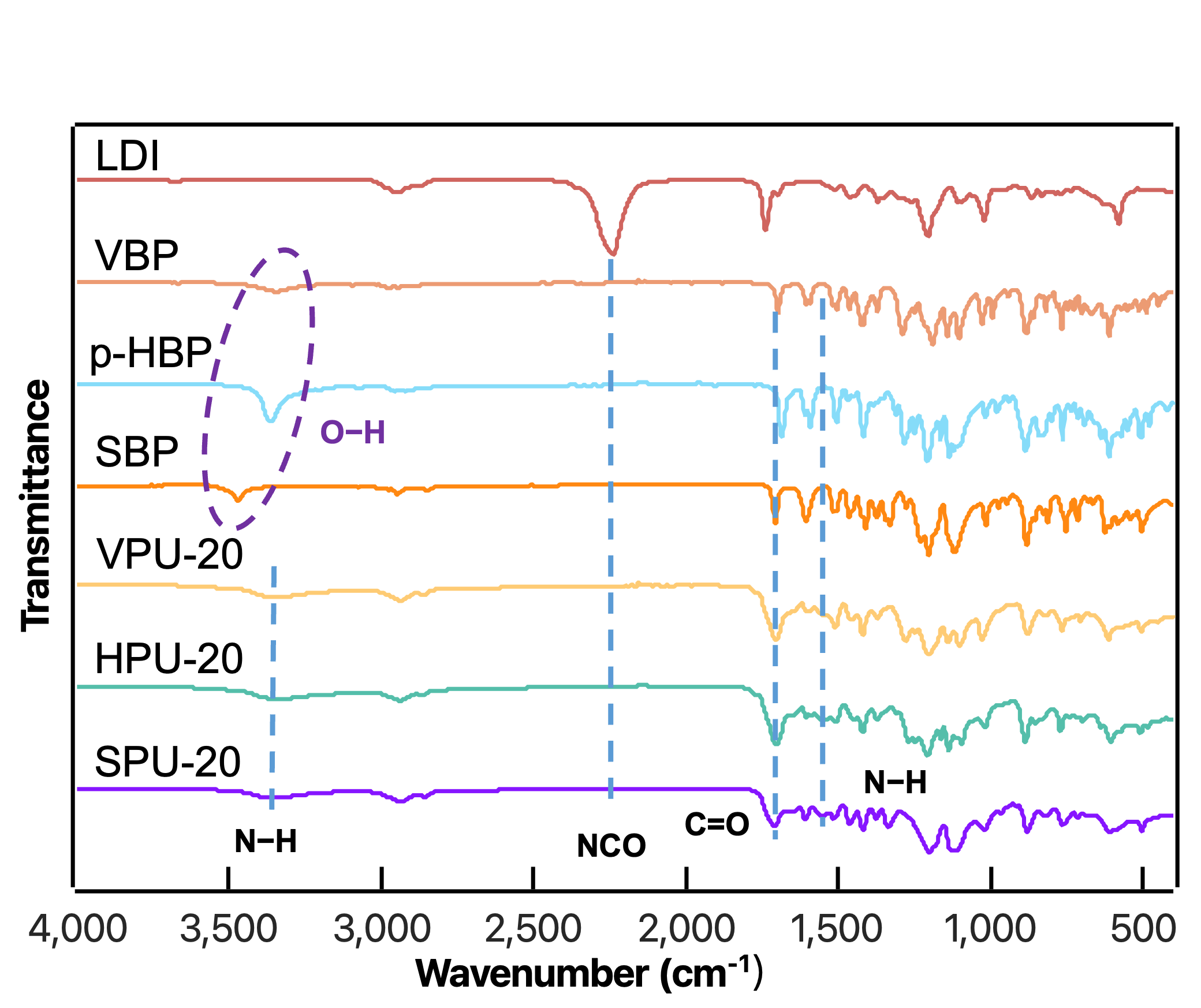


### Figure S11. Fourier Transform Infrared spectra (FTIR) of VBP, *p*-HBP, SBP, LDI and XPU-20.


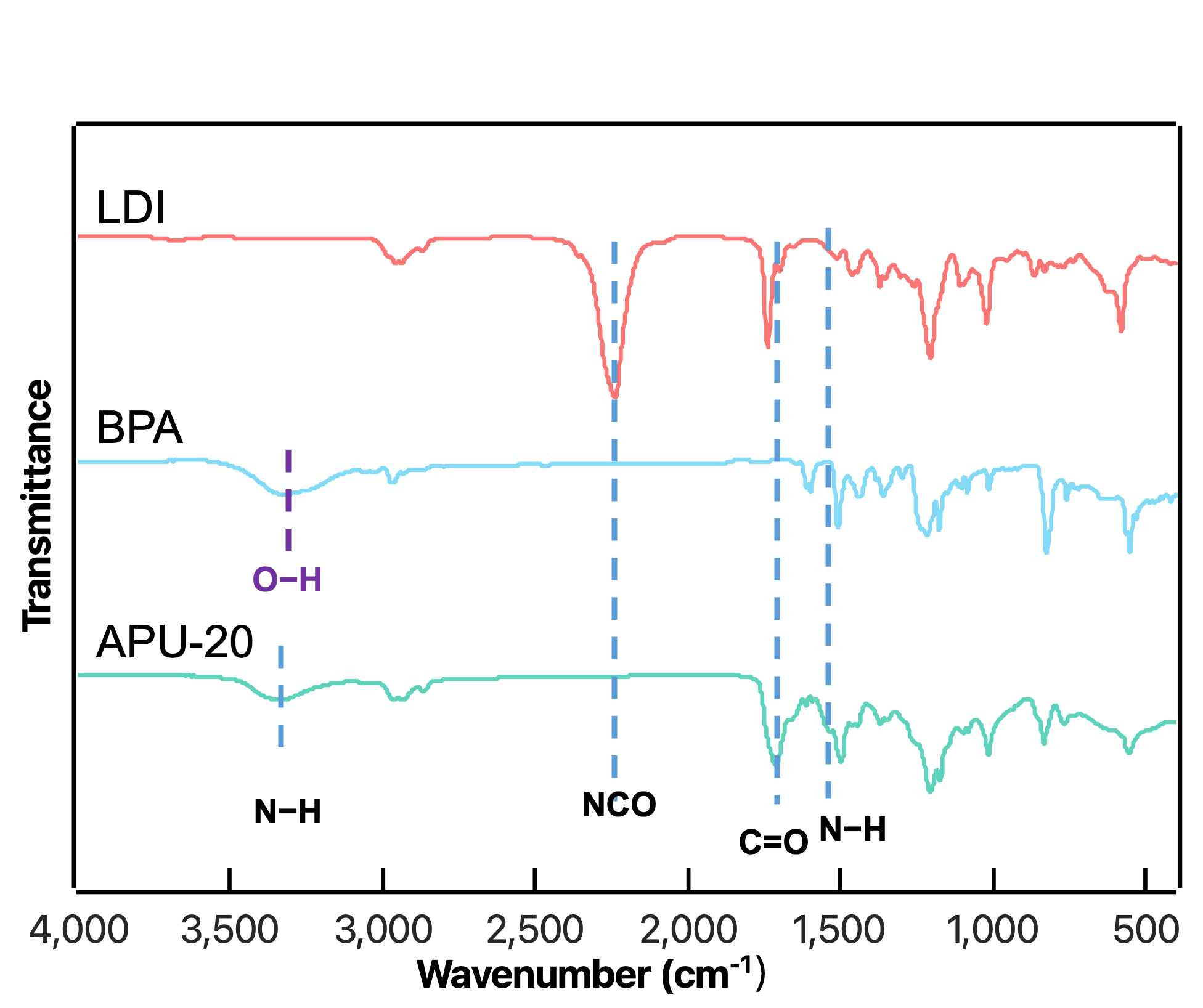


### Figure S12. Fourier Transform Infrared spectra (FTIR) of BPA, LDI and APU-20.

## Gel Fraction Test


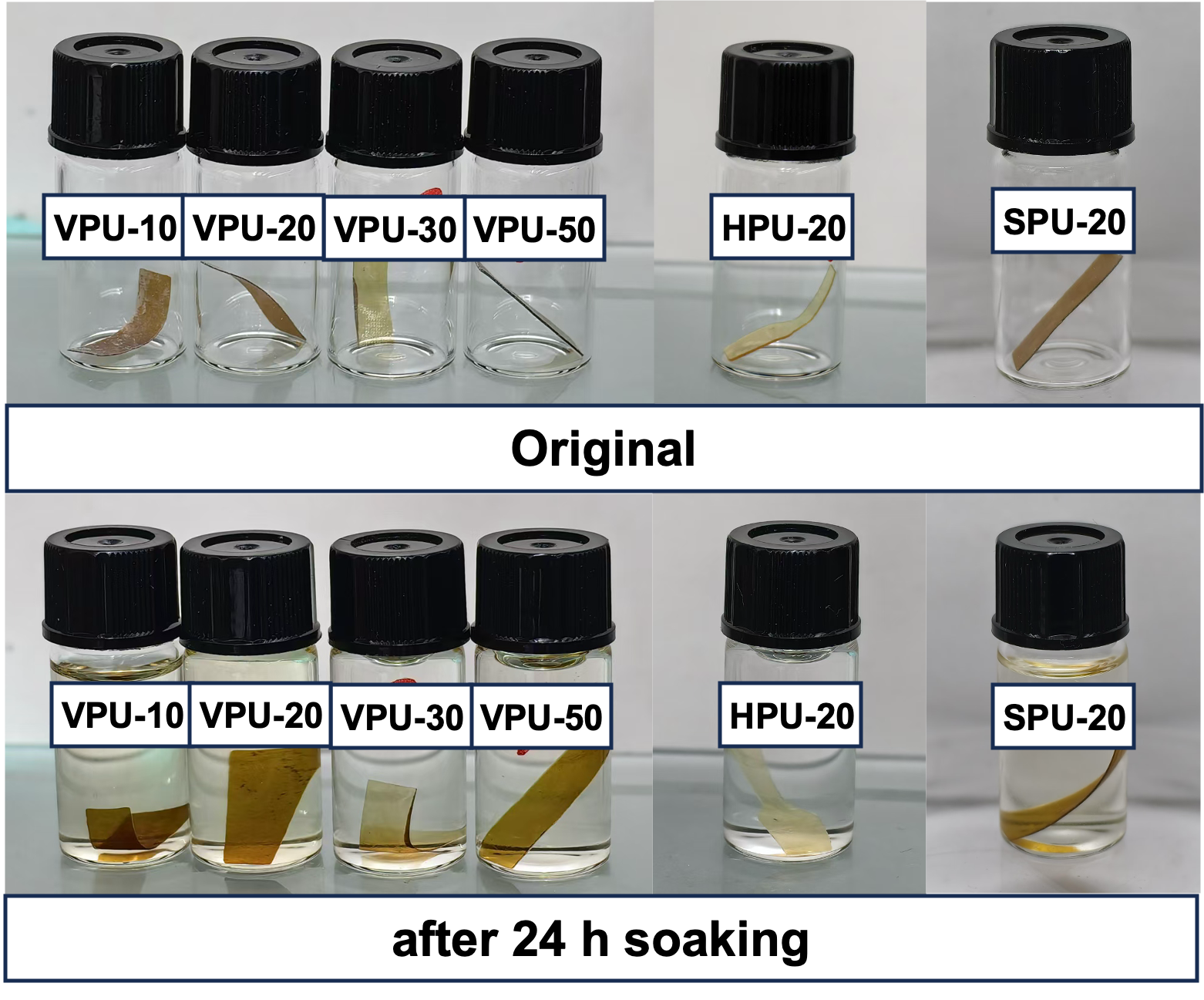


### Figure S13. Gel fraction test of Bio-based renewable PUs.

### Table S2. Gel Fraction Test of Bio-Based Renewable PUs

| Sample | VPU-10 | VPU-20 | VPU-30 | VPU-50 | HPU-20 | SPU-20 |
| --- | --- | --- | --- | --- | --- | --- |
| Gel fraction (%) | 89.2 | 92.4 | 93.8 | 94.3 | 93.3 | 93.8 |

## Dynamic Dissociation Properties of Linear Model Compounds

###
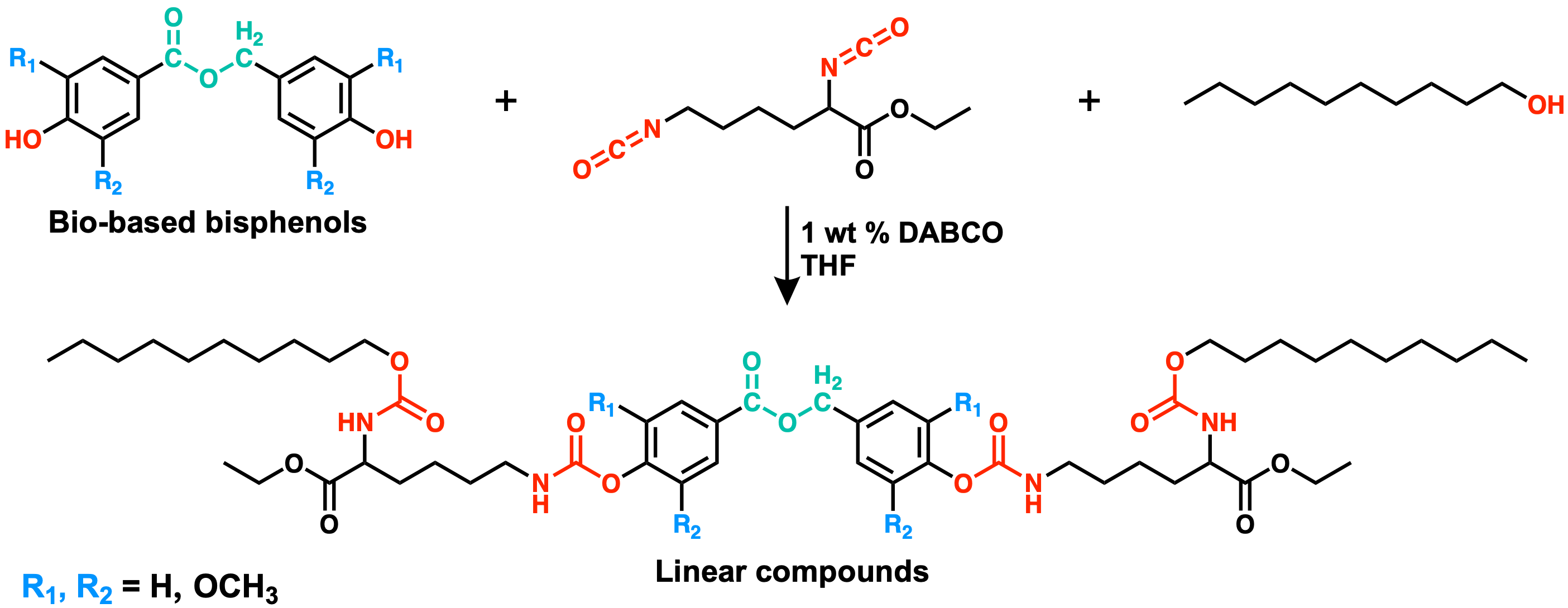
Figure S14. Synthesis route of linear compounds for *in-situ* ATR-IR detection.


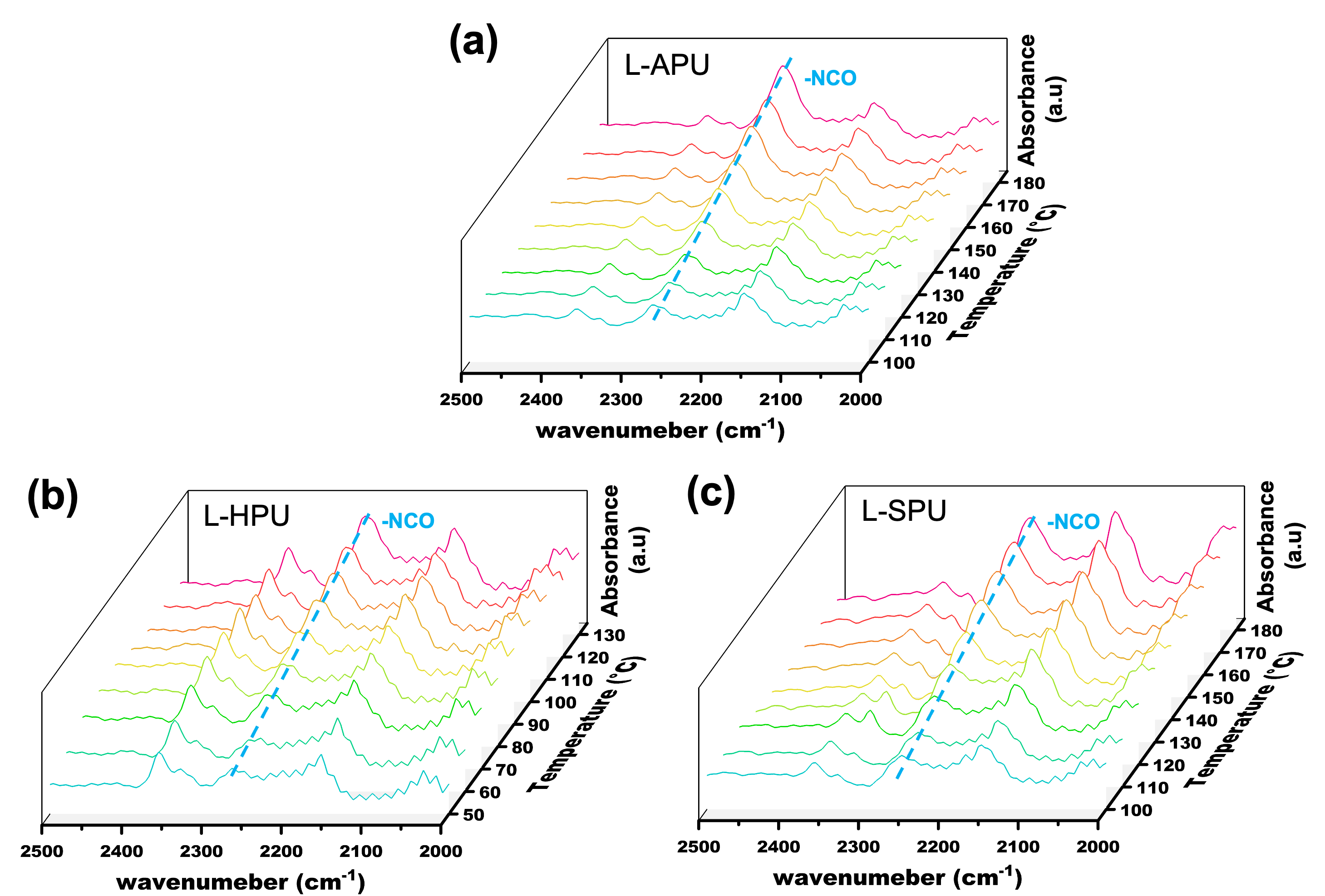


### Figure S15. The temperature-dependent *in-situ* ATR-IR spectra of the dissociative −NCO of the linear model compounds : (a)L-APU and (b)L-HPU and (c)L-SPU.

## Dynamic Exchange Properties of Small Molecule Model Compounds


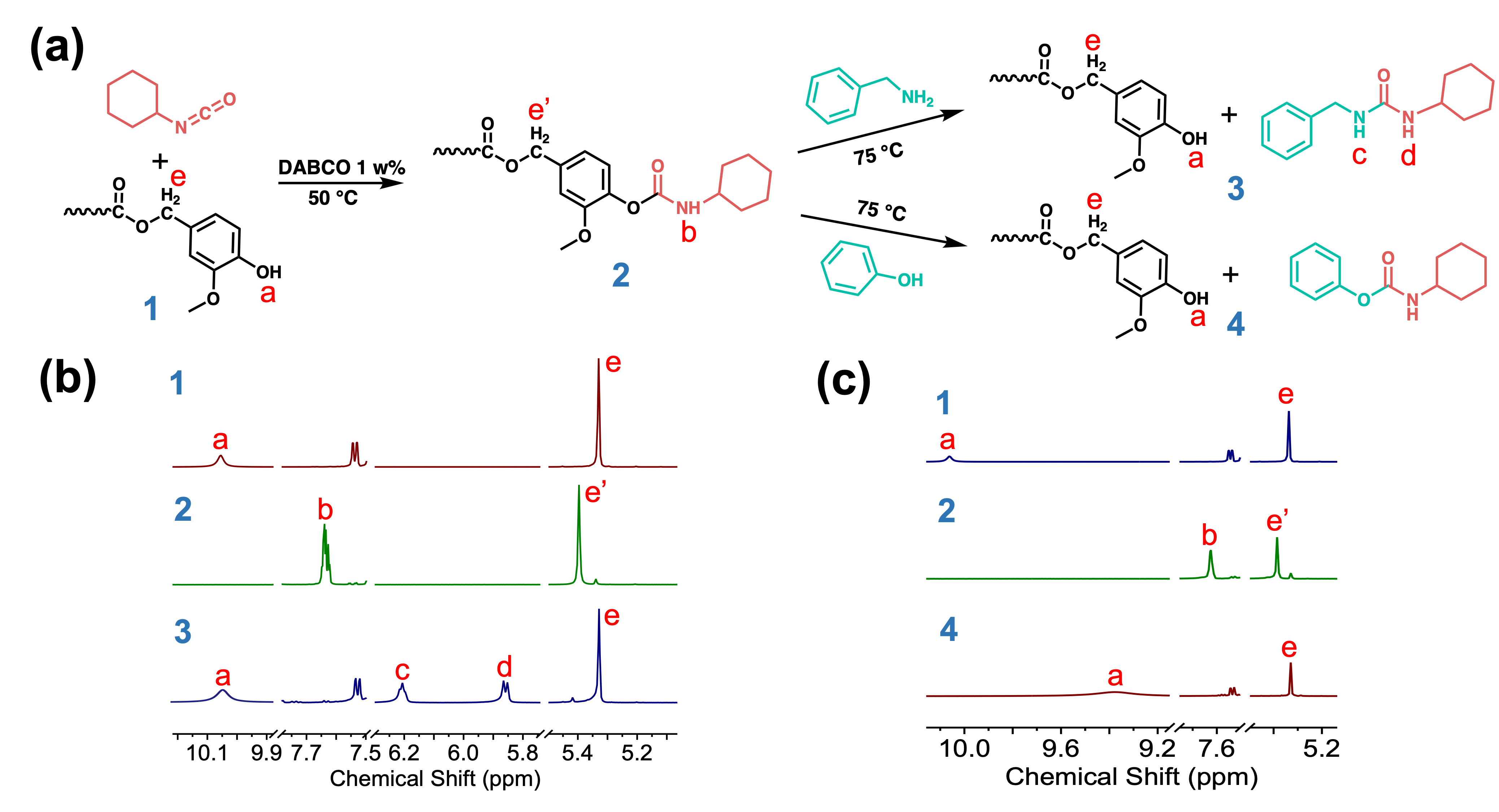


### Figure S16. The phenol-carbamate dynamic exchange of the small molecular model compound. (a) Reactions of VBP, CHI and BA/Phenol. ^1^H NMR spectra of the stock solution of the (b) step **1**, **2**, **3** and (c) step **1**, **2**, **4** in DMSO-*d*_6_.

## Thermal and Mechanical Properties


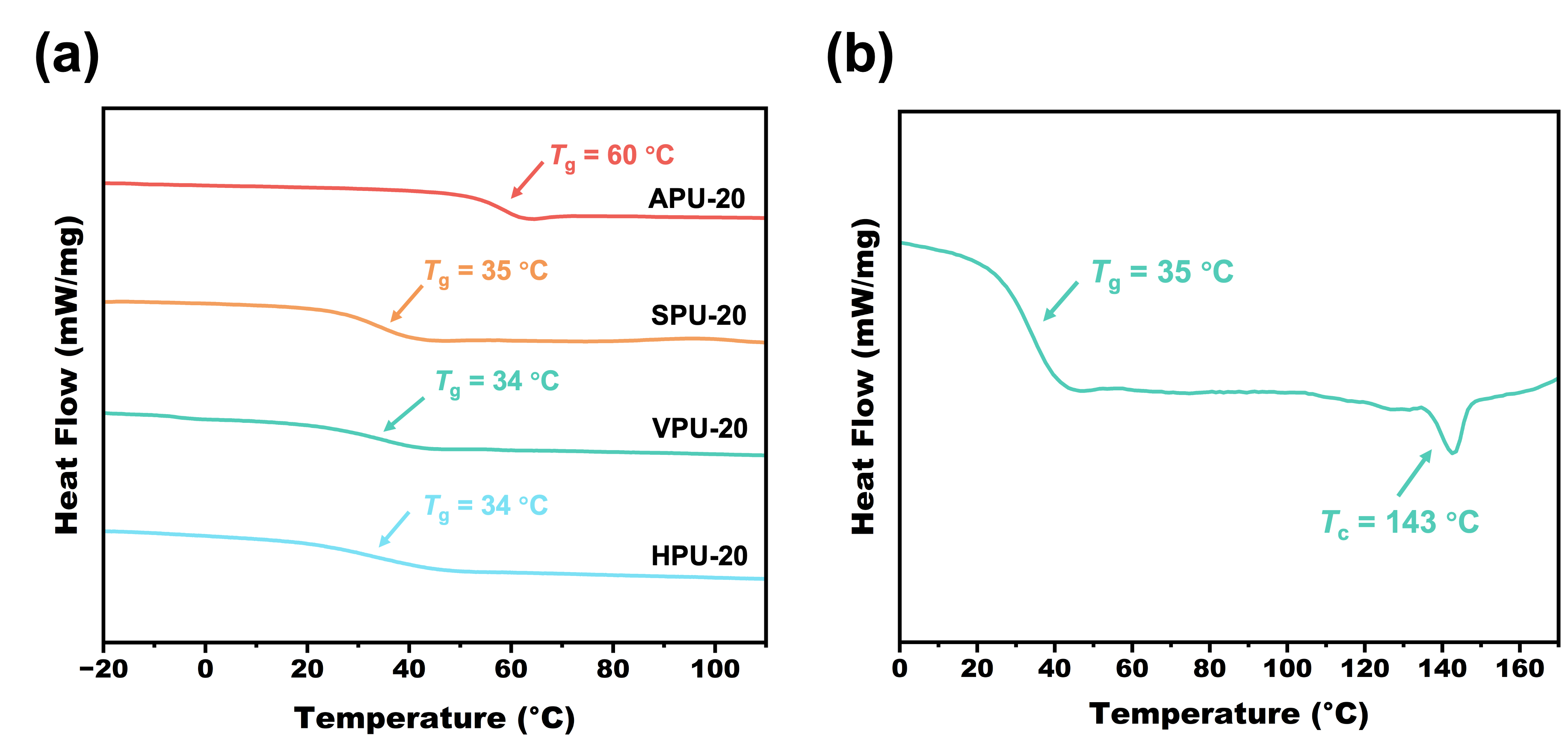


### Figure S17. (a) DSC curves of XPU-20. (b) DSC curves of SPU-20. The third heating curve was analyzed to obtain the *T*_g_ value of all samples.


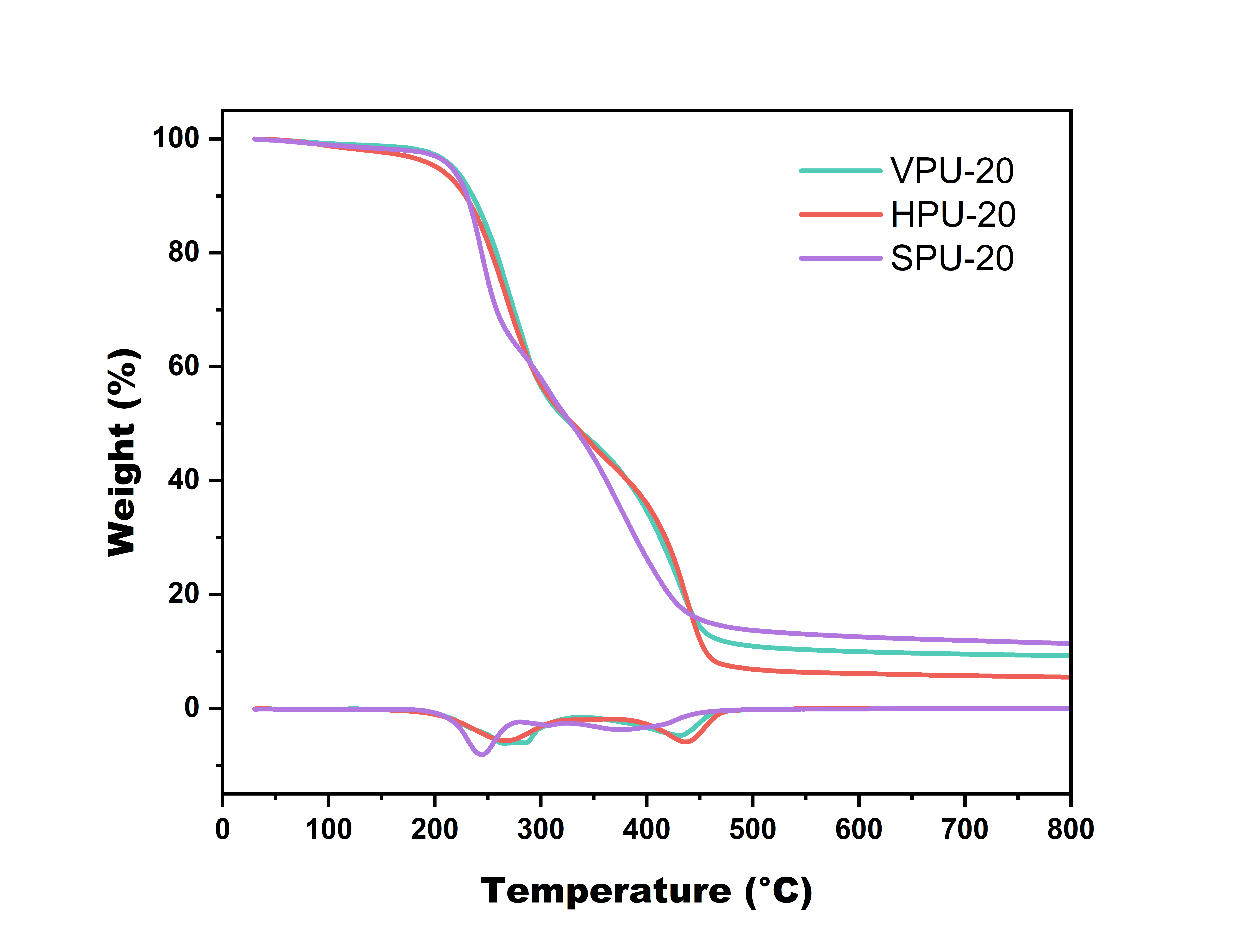


### Figure S18. TGA and DTG curves of XPU-20; heating rate: 10 °C/min; atmosphere: N_2_.


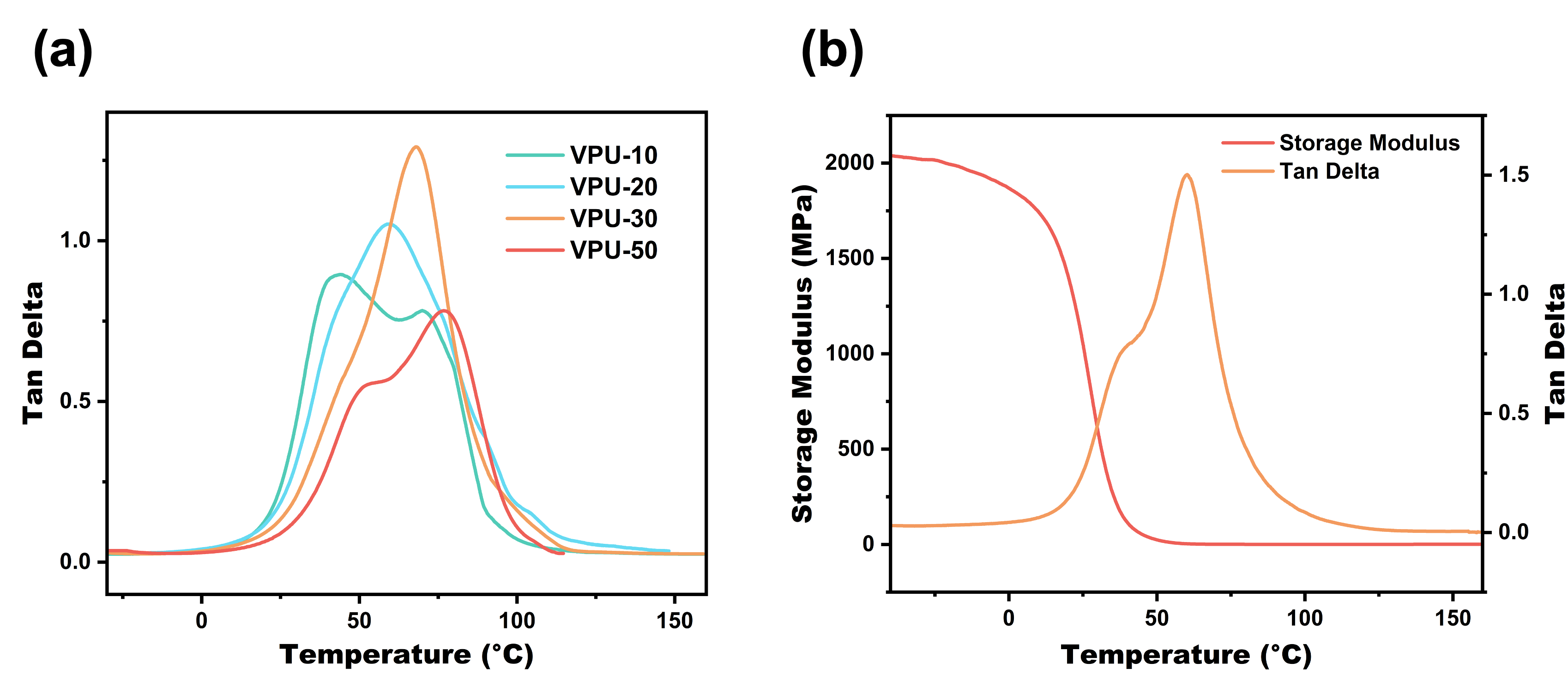


### Figure S19. (a) Tan δ curves of VPU-*p*. (b) Storage modulus and tan δ curves of HPU-20.

## Thermal Processing Recyclable Properties


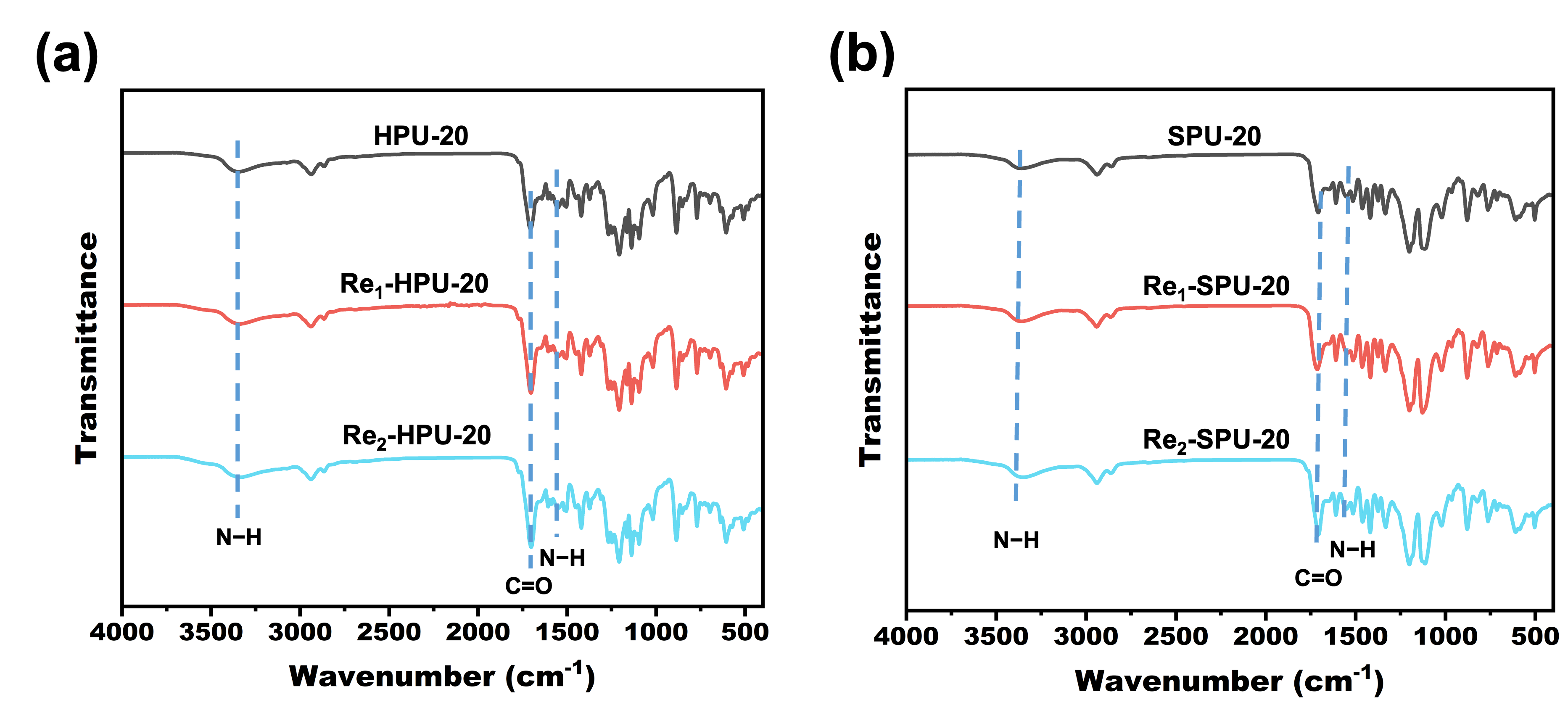


### Figure S20. The FTIR spectra of the original and reprocessed (a) HPU-20 and (b) SPU-20.

## Shape Memory and Cyclic Properties


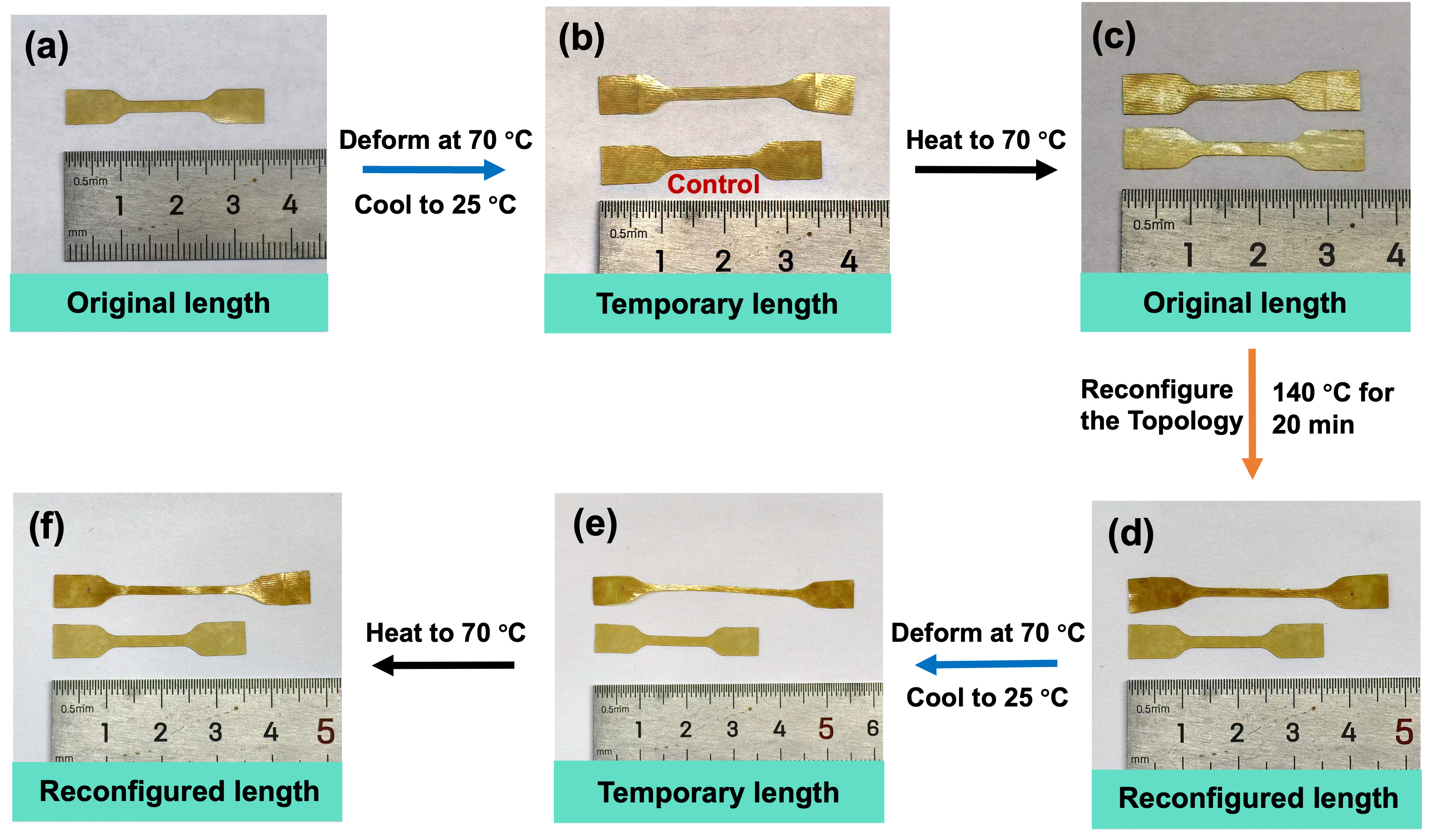


### Figure S21. The shape memory and shape reconfiguration processes of VPU-20 specimen under the CANs.


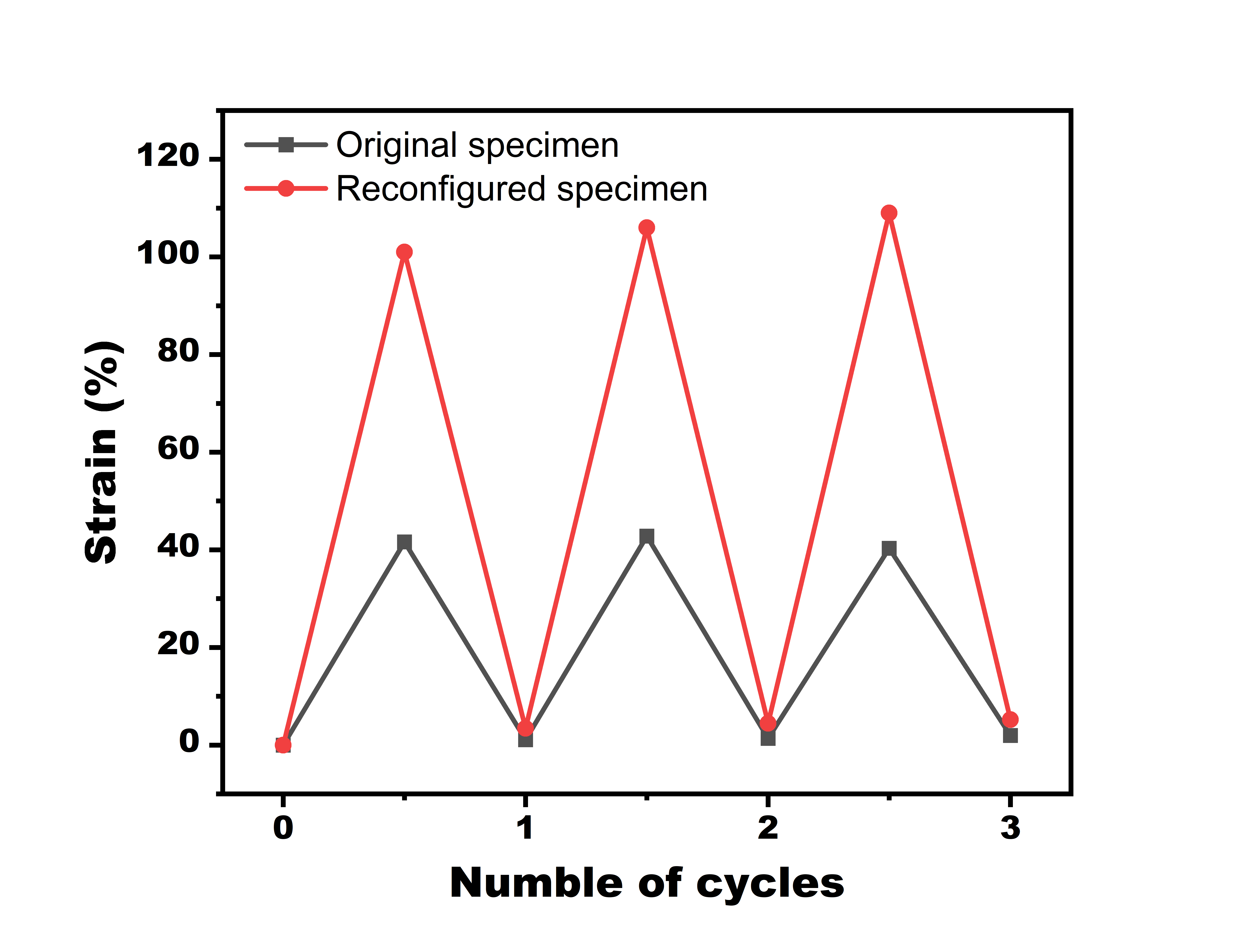


### Figure S22. Shape memory cycle experiments on the stretching of original and reconfigured specimens. Each cycle consists of hot stretching, cooling to fix the shape, and reheating for shape recovery. Detailed experimental procedures are provided in the experimental section.

### Table S3. Recovery Ratio of Shape Memory Cycles for Original and Reconfiguration Specimens

| Sample | Recovery_1_*^a^* ratio (%) | Recovery_2_ ratio (%) | Recovery_3_ ratio (%) | Average recovery ratio (%) |
| --- | --- | --- | --- | --- |
| VPU-20 | 97.4 | 96.8 | 95.4 | 96.5 |
| Reconfigured-VPU-20 | 96.6 | 95.8 | 95.1 | 95.8 |

*^a^* The numerical subscript represents the number of cycles.

## Degradation Properties


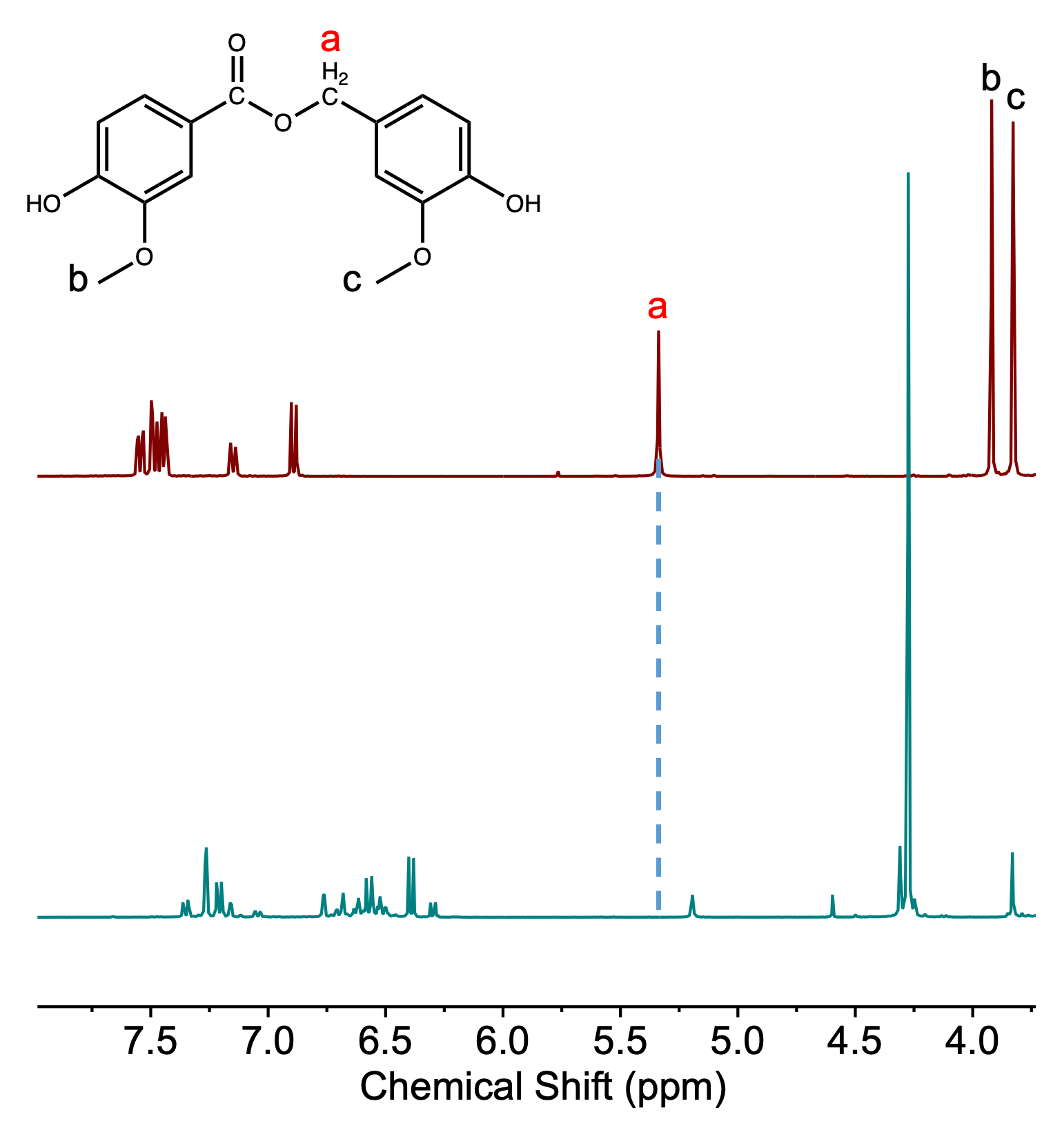


### Figure S23. ^1^H NMR spectra of VBP degradation in a 0.1 M NaOD solution (DMSO-*d*₆/D₂O = 1:1, v/v).


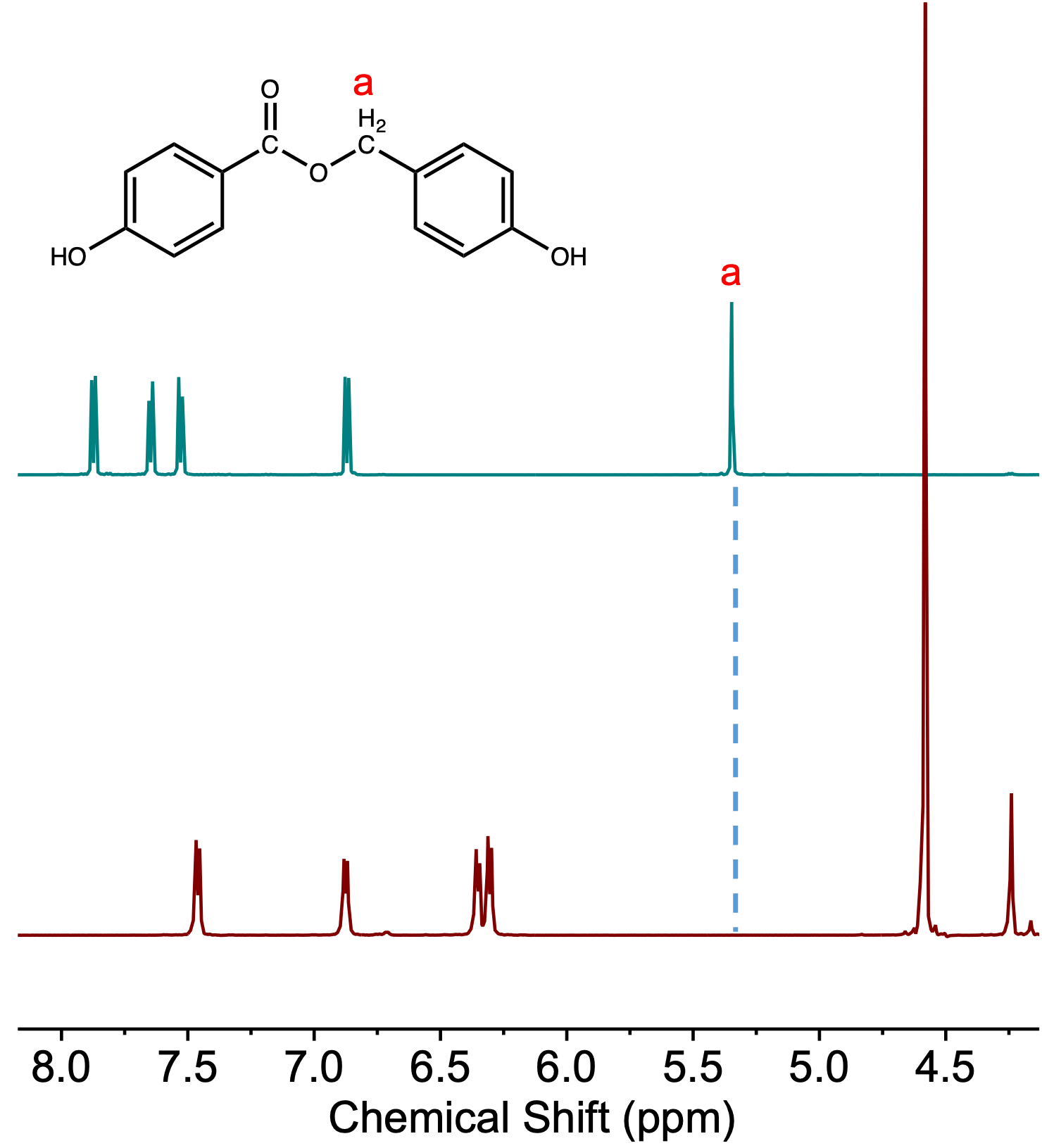


### Figure S24. ^1^H NMR spectra of *p*-HBP degradation in a 0.1 M NaOD solution (DMSO-*d*₆/D₂O = 1:1, v/v).


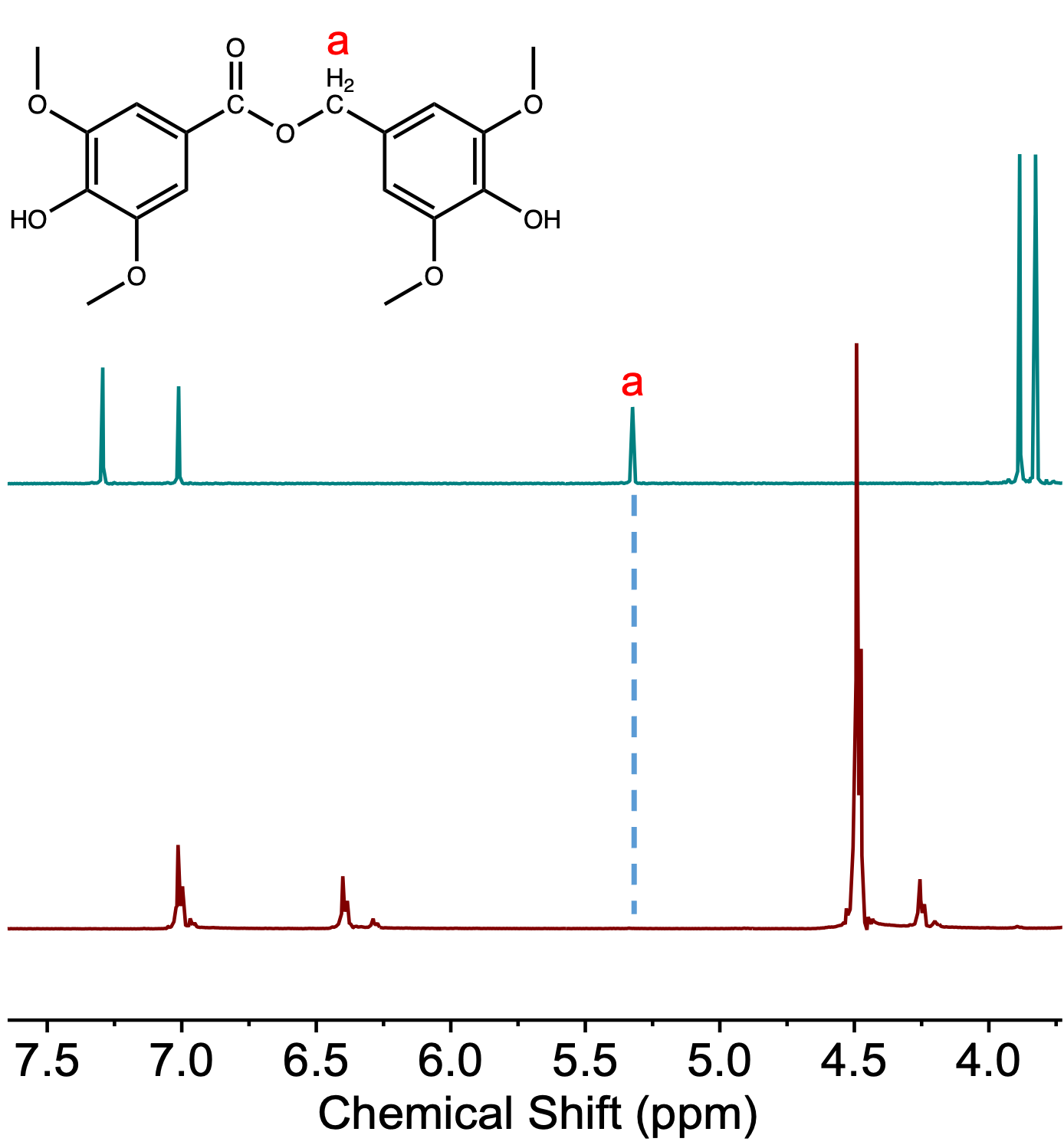


### Figure S25. ^1^H NMR spectra of SBP degradation in a 0.1 M NaOD solution (DMSO-*d*₆/D₂O = 1:1, v/v).


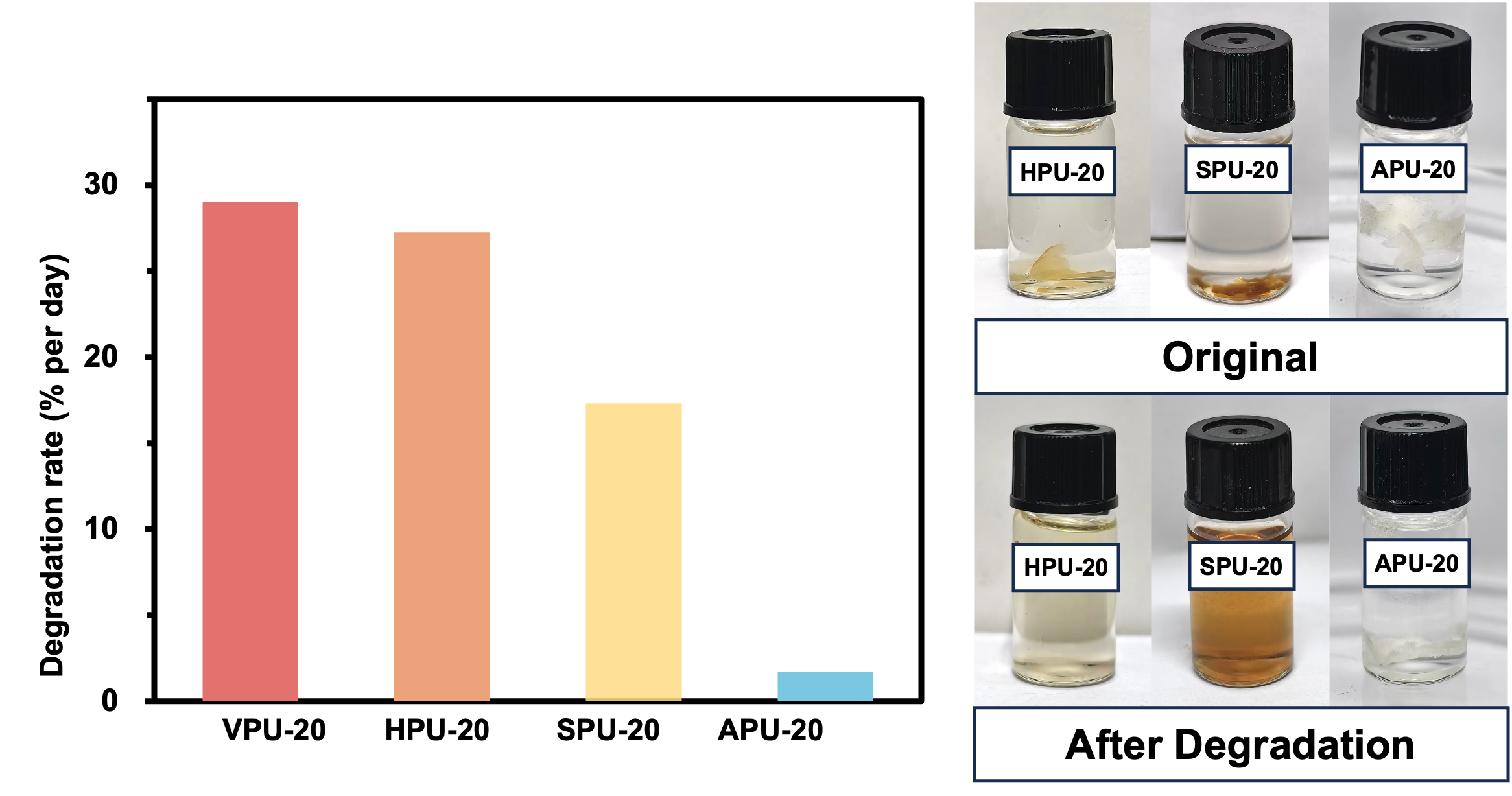


### Figure S26. The bar chart of the degradation rate of XPU-20 (X = V, A, H and S) in 0.1 M NaOH solutions (THF/H₂O = 1:1, v/v).
